# Three distinct biochemical subtypes of C_4_ photosynthesis? A modelling analysis

**DOI:** 10.1093/jxb/eru058

**Published:** 2014-03-08

**Authors:** Yu Wang, Andrea Bräutigam, Andreas P. M. Weber, Xin-Guang Zhu

**Affiliations:** ^1^State Key Laboratory for Hybrid Rice, CAS-MPG Partner Institute for Computational Biology, Shanghai Institutes for Biological Sciences, Chinese Academy of Sciences, Shanghai 200031, China; ^2^Key Laboratory of Computational Biology, CAS-MPG Partner Institute for Computational Biology, Shanghai Institutes for Biological Sciences, Chinese Academy of Sciences, Shanghai 200031, China; ^3^Institute of Plant Biochemistry, Cluster of Excellence on Plant Sciences (CEPLAS), Heinrich-Heine University, 40225 Düsseldorf, Germany

**Keywords:** Efficiency, flexibility, mixture, NADP-ME, NAD-ME, PEPCK.

## Abstract

C_4_ photosynthesis has higher light-use, nitrogen-use, and water-use efficiencies than C_3_ photosynthesis. Historically, most of C_4_ plants were classified into three subtypes (NADP-malic enzyme (ME), NAD-ME, or phosphoenolpyruvate carboxykinase (PEPCK) subtypes) according to their major decarboxylation enzyme. However, a wealth of historic and recent data indicates that flexibility exists between different decarboxylation pathways in many C_4_ species, and this flexibility might be controlled by developmental and environmental cues. This work used systems modelling to theoretically explore the significance of flexibility in decarboxylation mechanisms and transfer acids utilization. The results indicate that employing mixed C_4_ pathways, either the NADP-ME type with the PEPCK type or the NAD-ME type with the PEPCK type, effectively decreases the need to maintain high concentrations and concentration gradients of transport metabolites. Further, maintaining a mixture of C_4_ pathways robustly affords high photosynthetic efficiency under a broad range of light regimes. A pure PEPCK-type C_4_ photosynthesis is not beneficial because the energy requirements in bundle sheath cells cannot be fulfilled due to them being shaded by mesophyll cells. Therefore, only two C_4_ subtypes should be considered as distinct subtypes, the NADP-ME type and NAD-ME types, which both inherently involve a supplementary PEPCK cycle.

## Summary

Only two distinct C_4_ subtypes exist: the NADP-malic enzyme and NAD-malic enzyme subtypes. Both inherently involve a supplementary phosphoenolpyruvate carboxykinase cycle, which affords increased metabolic flexibility and robustness under diverse environments.

## Introduction

Plants using C_4_ photosynthesis have higher potential energy-conversion efficiency than C_3_ plants because of a CO_2_-concentrating mechanism that largely reduces photorespiration (Zhu *et al.*, 2008; Amthor, 2010). In most cases, with the exception of single-cell C_4_ photosynthesis (Edwards *et al.*, 2004), this CO_2_-concentrating mechanism usually requires compartmentalized photosynthetic reactions into two distinct cell types: bundle sheath cells (BSCs) and mesophyll cells (MCs) (Hatch, 1987). In C_4_ plants, CO_2_ is fixed by phosphoenolpyruvate (PEP) carboxylase (PEPC) in the MC cytosol, and the resulting C_4_ acid is subsequently converted to either malate or aspartate. Then, the C_4_ acid is transported to the BSCs where it is decarboxylated to release CO_2_ around Rubisco. Decarboxylation in BSCs, together with the CO_2_ diffusion barrier between BSCs and MCs, elevates the CO_2_ concentration around Rubisco, thereby minimizing photorespiration (von Caemmerer and Furbank, 2003).

Traditionally, C_4_ photosynthesis is classified into three biochemical subtypes, according to their different decarboxylation mechanisms. The first enzyme of the C_4_ cycle, PEPC, is common to all three subtypes. The product of PEPC, oxaloacetate (OAA), can be either reduced to malate by malate dehydrogenase (MDH) or converted to aspartate (Asp) by aspartate aminotransferase (Hatch and Slack, 1966; Hatch *et al.*, 1975; Pick *et al.*, 2011). Malate is then transported to BSCs in NADP-malic enzyme (ME) subtype plants while Asp is transported in NAD-ME and PEPCK types (Hatch *et al.*, 1975; Kanai and Edwards, 1999; Furbank, 2011). In the NADP-ME type, malate is used to generate pyruvate and CO_2_, with the formation of NADPH catalysed by NADP-ME. In the other two decarboxylation types, aspartate is converted back to OAA in BS cytosol or mitochondria (Taniguchi *et al.*, 1995). In the NAD-ME type, OAA is reduced to malate by MDH, and the NAD-ME then catalyses splitting of malate to release CO_2_ and pyruvate in mitochondria. In the PEP-carboxykinase (PEPCK) type, most of the OAA is converted to PEP and CO_2_ in BSC cytosol by PEPCK. NAD-ME is used to provide NADH for generation of ATP, which can be used to fuel PEPCK (Kanai and Edwards, 1999). NAD-ME can also balance the amino groups between MCs and BSCs via the return of alanine to MCs (Furbank, 2011). Since Asp brings an amino group from MCs to BSCs, OAA generated from Asp can be utilized in two ways. One is that PEPCK generates PEP, which directly returns to MCs without an amino group; the other is that OAA is converted to malate, which is then decarboxylated to pyruvate by NAD-ME. The resulting pyruvate can be converted to alanine and returned to MCs, thereby transporting the amino group.

Although C_4_ photosynthesis has long been classified into three distinct subtypes, multiple lines of evidence suggest that some flexibility in C_4_ photosynthetic pathways exists in the same leaf: specifically that NADP-ME and PEPCK subtypes might coexist; similarly, NAD-ME type and PEPCK subtypes can also coexist. Early ^14^C-labelling experiments in maize indicated that radioactively labelled carbon provided as CO_2_ is mostly incorporated into malate, but also to a substantial degree into aspartate, a compound normally considered as not present in the classical NADP-ME-type model (Hatch, 1971). Later, Chapman and Hatch (1981) showed that isolated BSCs of maize can use aspartate and oxoglutarate to produce CO_2_ and Pick *et al.* (2011) showed that maize leaves contain sufficient activities of the aminotransferases to carry the required flux. Furthermore, a similar phenomenon has also been found in *Flaveria bidentis*, an NADP-ME dicot species (Meister *et al.*, 1996). Key C_4_ enzyme activities also indicate that the ratio of asparate to malate transferred varies in NADP-ME type species (Kanai and Edwards, 1999). The flexibility of C_4_ subtypes has also been shown through coexistence of key enzymes. For example, in maize, PEPCK is present, active, and capable of supporting high rates of aspartate-dependent photosynthesis in isolated BSCs (Walker *et al.*, 1997; Wingler *et al.*, 1999; Majeran *et al.*, 2010; Pick *et al.*, 2011). The PEPCK transcript is expressed at high levels in maize BSCs (Furumoto *et al*., 1999, 2000). Also in the NAD-ME species *Cleome gynandra*, high PEPCK activity was detected (Sommer *et al.*, 2012) and different C_4_ dicots also contain PEPCK in addition to the major decarboxylation enzyme (Muhaidat *et al.*, 2007; Muhaidat and McKown, 2013).

The flexibility or coexistence of different C_4_ subtypes, i.e. between NADP-ME and PEPCK subtypes, or between NAD-ME and PEPCK subtypes, is also reflected in the theoretical quantum yield and observed energy-conversion efficiencies between plants considered having different subtypes. Theoretical considerations on quantum yield and leakiness of CO_2_ from BSCs suggested that the efficiency of C_4_ subtypes should be different and that the PEPCK subtype has the highest energy-conversion efficiency (Hatch, 1995; von Caemmerer and Furbank, 1999; von Caemmerer and Furbank, 2003). However, experimental evidence for this is still insufficient (Ghannoum *et al.*, 2001; Furbank, 2011).

Considering these facts, it is highly likely that the so-called C_4_ subtypes actually coexist in C_4_ plants. So far, the physiological significance of this potential coexistence of C_4_ subtypes is inadequately studied. This study uses a systems modelling approach to theoretically evaluate the potential physiological consequences of having different mixtures of C_4_ subtypes and further discusses whether the PEPCK pathway should indeed be considered as an independent or as a supplementary pathway.

## Materials and methods

### Model development

**Fig. 1.**
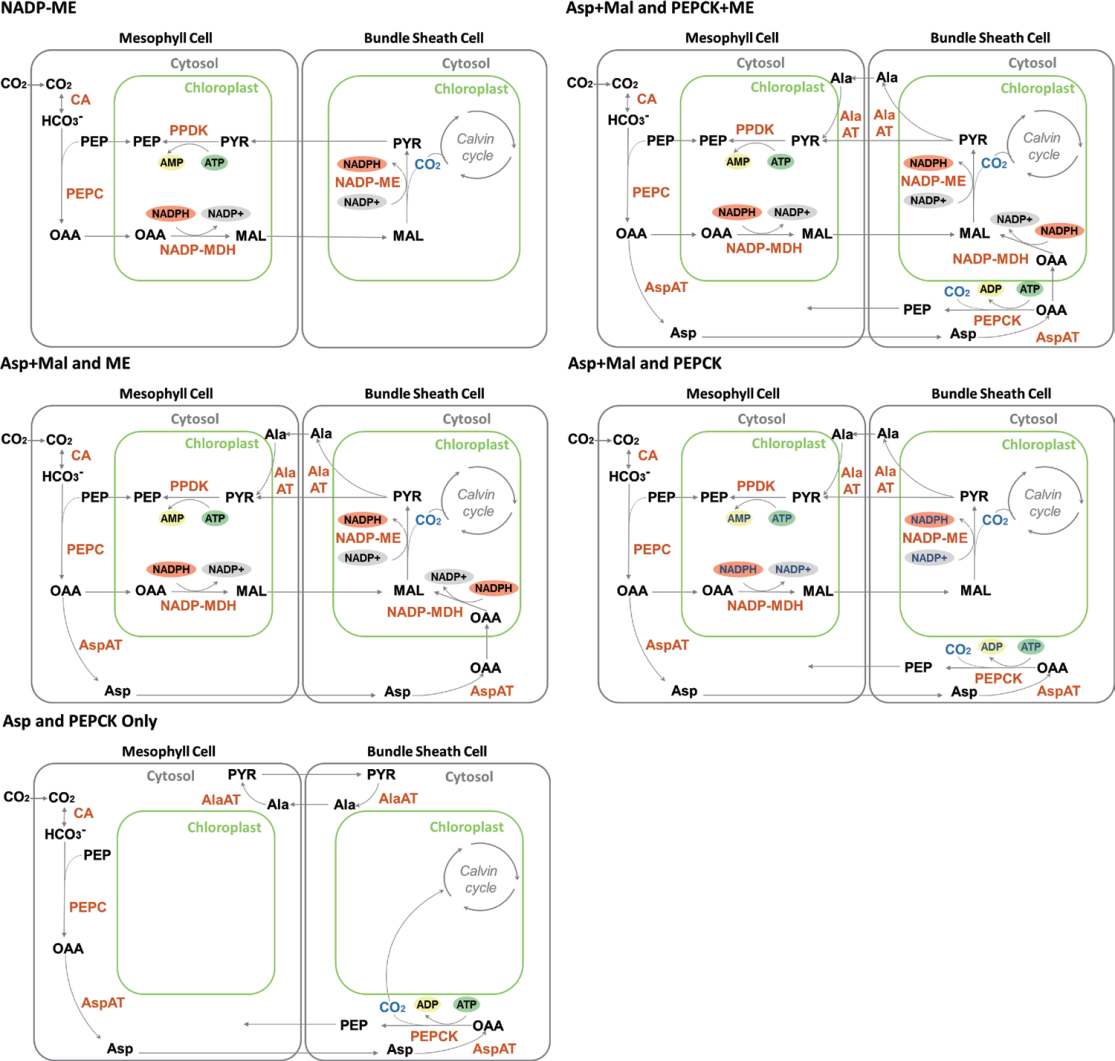
Models simulating different combinations of C_4_ pathways. Dark orange letters indicate enzymes and black letters indicate metabolites. AlaAT, alanine aminotransferase; AspAT, aspartate aminotransferase; MAL, malate; NADP-MDH, NADPH-malate dehydrogenase; NADP-ME, NADP-malic enzyme; OAA, oxaloacetate; PEP, phosphoenolpyruvate; PEPC, phosphoenolpyruvate carboxylase; PEPCK, phosphoenolpyruvate carboxykinase; PPDK, pyruvate phosphate dikinase; PYR, pyruvate.

The models developed here are depicted diagrammatically in Fig. 1. The individual models were developed following the basic procedure as in Zhu *et al.* (2007). Essentially, after the reaction diagrams were established, differential equations, rate equations, and algebraic equations representing conserved quantities were developed. The models were implemented in MATLAB and solved using *ode15s*.

With the models, this work computed changes in the metabolite concentrations (dM/dt) by the differences between rates of fluxes generating and consuming this metabolite:

$$\frac{dM}{dt}=v_{in}-v_{out}$$ (1)

where *v*_*in*_ and *v*_*out*_ represent the rate of fluxes generating and consuming metabolite (M), respectively. This work developed one differential equation as equation 1 for each metabolite used in the model. The equations and parameters used in the model were listed in Table 1 and the Supplementary Data (available at *JXB* online). The C_4_ NADP-ME type model (Wang *et al.*, 2014) was used as a basis of the kinetic models of mixed C_4_ pathways, upon which additional pathways were added to form various mixed-pathway models (Supplementary Fig. S1 available at *JXB* online).

**Table 1.** Enzyme abbreviations and maximum activities

EC^*a*^	Abbreviation	Full name	*V*_*max*_ (μmol m^–2^ s^–1^)	Reference
1.1.1.40	MDH	Malate dehydrogenase (NADP+)	90	Kanai and Edwards (1999); Hatch (1987)
1.1.1.82_B_	MDH	Malate dehydrogenase (NADP+)	60^*b*^ ^,*c*^	
1.1.1.82	PPDK	Pyruvate phosphate dikinase	90	Kanai and Edwards (1999); Hatch (1987)
2.6.1.1_B_	AspAT	Aspartate transaminase	400^*b*^	Pick *et al.* (2011)
2.6.1.1_M_	AspAT	Aspartate transaminase	400^*a*^	Pick *et al.* (2011)
2.6.1.2_B_	AlaTA	Alanine transaminase	400^*b*^	Pick *et al.* (2011)
2.6.1.2_M_	AlaTA	Alanine transaminase	400^*b*^	Pick *et al.* (2011)
2.7.9.1	NADP-ME	NADP-malic enzyme	90	Kanai and Edwards (1999); Hatch (1987)
4.1.1.31	PEPC	Phosphoenolpyruvate carboxylase	170	Kanai and Edwards (1999); Hatch (1987); von Caemmerer (2000)
4.1.1.39	Rubisco	Ribulose-bisphosphate carboxylase	65	Kanai and Edwards (1999); Hatch (1987)
4.1.1.49	PEPCK	Phosphoenolpyruvate carboxykinase	60^*b*^	Pick *et al.* (2011)

^*a*^Subscripts M and B indicate enzyme allocation in mesophyll and bundle sheath cells, respectively, and thus distinguish enzymes localized in both.

^*b*^The values are assumed.

^*c*^It is not clear whether MDH in BSC also take part in C_4_ photosynthesis; therefore, no reference can be provided.

### Combinations of C_4_ pathways

Experimental evidence has suggested that the so-called three C_4_ subtypes can coexist with each other (Hatch, 1971; Chapman and Hatch, 1981; Meister *et al.*, 1996; Pick *et al.*, 2011). For example, in *Zea mays*, about 25% of the initial carbon label partitions to aspartate and about 75% to malate (MAL), which shows that *Z. mays* uses both as transfer acids (Hatch, 1971). This mixture of C_4_ subtypes were also clearly demonstrated in a recent transcriptomics and enzyme activity study (Pick *et al.*, 2011). The use of amino acids in addition to malate and pyruvate does not necessarily require the presence of PEPCK as the decarboxylation enzyme, as demonstrated in *Sorghum bicolor*, which does not have PEPCK but transcriptome data shows high level of Asp transaminase and Ala transaminase expression which suggest amino acids are also used as transfer acids (Gutierrez *et al.*, 1974). Based on the knowledge above, the current work developed different systems models representing various combinations of C_4_ subtypes. Those pathways with combinations of C_4_ subtypes were termed as mixed C_4_ pathways, and models representing these mixed pathways were termed as mixed-pathway models. The models were named after the transfer C_4_ acid and the decarboxylase used: Asp+MAL and PEPCK model, Asp+MAL and ME model, and Asp+MAL and PEPCK+ME model. In the Asp+MAL and PEPCK model, the transferred aspartate is decarboxylated in BSC cytosol by PEPCK, while in the Asp+MAL and ME model, the transferred aspartate is decarboxylated in BSC chloroplasts by NADP-ME. The Asp+MAL and ME model requires existence of MDH in BSCs. The Asp+MAL and PEPCK+ME model can be regard as an integration of the other two models. In the hypothetical Asp and PEPCK-only model, aspartate is the only transfer C_4_ acid and PEPCK is the only decarboxylase in C_4_ cycle.

### Light reactions

The electron transport rate (*J*) is used to calculate the rates of ATP and NADPH synthesis in the models. The maximum rate of ATP and NADPH synthesis reactions were described as:

$$V_{\max}=\min(V_{\max E},\;V_{\max J})$$ (2)

where *V*_*maxE*_ is the maximum rate of ATP and NADPH synthesis determined by the properties of ATP synthase and NADP^+^ reductase, and *V*_*maxJ*_ is calculated by the electron transport rate:

$$V_{\max J}=\varepsilon \cdot J$$ (3)

where ε is the ATP/e^–^ ratio or NADPH/e^–^ ratio.

A biochemical model (Ögren and Evans, 1993; von Caemmerer, 2000) was used to calculate *J*:

$$J=\frac{I_{2}+J_{\max}-\sqrt{{\left(I_{2}+J_{\max}\right)}^{2}-4\theta I_{2}J_{\max}}}{2\theta }$$ (4)

where *I*_*2*_ is the photosynthetic active radiation absorbed by photosystem II (PSII) which is calculated from the input of photosynthetic photon flux density (PPFD), *J*_*max*_ is the maximal electron transport rate, and θ is an empirical curvature factor (Evans, 1989; von Caemmerer, 2000).

*J* of MC (*J*_*m*_) and BSC (*J*_*b*_) were calculated separately (Wang *et al.*, 2014; Supplementary Data available at *JXB* online), In this model, the proportion of linear electron transport (LET) in BSCs, as reflected in the PSII content in BSCs, was assumed to be variable:

$$I_{l\_b}=\frac{1}{2}uI_{b}$$ (5)

$$J_{\max\_l\_b}=vJ_{\max\_b}$$ (6)

$$J_{l\_b}=\frac{I_{l\_b}+J_{\max\_l\_b}-\sqrt{{\left(I_{l\_b}+J_{\max\_l\_b}\right)}^{2}-4\theta I_{l\_b}J_{\max\_l\_b}}}{2\theta }$$ (7)

where *I_l_b_* and *J_maxl_b_* represent incident photosynthetic photon flux density and *J*_*max*_, which participate in liner electron transport in BSCs. *u* and *v* are variables representing the proportion of light and *J*_*max*_ of LET in BSCs; this model assumes that they are equal, i.e. *u*=*v*=LET_BSC_. If *u* and *v* are 0, there is no LET in BSCs; if *u* and *v* are 1, all light and *J*_*max*_ take part in LET in BSCs. *I*_*b*_ represents light absorbed by BSCs, and *J_max_b_* is the maximum electron transport capacity of BSCs. ATP used by PEPCK in BSCs cytosol is assumed to be supported by light reactions in BSCs.

O_2_ generation from LET was considered in both MCs and BSCs. The total equation is:

$$\begin{array}{c} \;H_{2}O+\;NADP^{+}+2ADP+2Pi\\ \to NADPH\;+\;2ATP\;+\frac{1}{2}O_{2}+H^{+} \end{array}$$ (8)

The reaction rate of equation 8 (*v*_*LET*_) was considered as:

$$v_{LET}=\frac{1}{2}J_{l}$$ (9)

where *J*_*l*_ is the LET rate. The LET rate of BSCs (*J_l_b_*) is calculated by equation 7. In MCs, the model assumed that all electrons were transported through the LET chain.

### CO_2_ assimilation rate

During the simulation, it was assumed that the model reached a steady state when the predicted metabolite concentrations did not change with time any more. The steady-state metabolite levels and flux rates were extracted from model output after the model reached a steady state. The CO_2_ assimilation rate (*A*) was calculated as (Farquhar *et al.*, 1980):

$$A=v_{c}-0.5v_{o}-R_{d}$$ (10)

where *v*_*c*_ and *v*_*o*_ are the rates of RuBP carboxylation and oxygenation, respectively, and *R*_*d*_ is the rate of mitochondrial respiration. The default value for *R*_*d*_ is 1 μmol m^–2^ s^–1^, with the rate of respiration in both BSCs (*R*_*b*_) and MCs (*R*_*m*_) being equal as 0.5 μmol m^–2^ s^–1^.

## Results

### Model structures and predicted photosynthetic rates under different light and CO_2_ levels

**Fig. 2.**
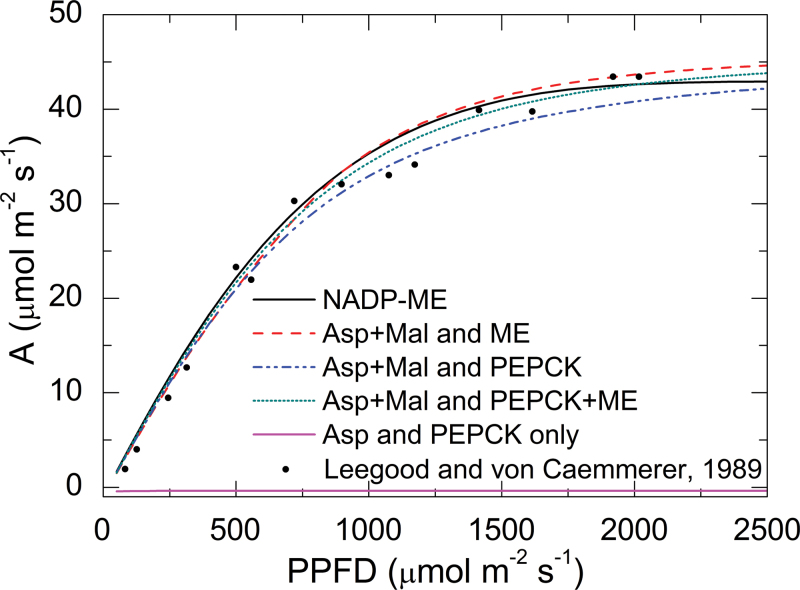
Curves for models predicting CO_2_ assimilation vs. photosynthetic photon flux density (PPFD). The models were named after the transfer C_4_ acid and the decarboxylase used. In the Asp+MAL and PEPCK model, the transferred aspartate is decarboxylated in BSC cytosol by PEPCK; in the Asp+MAL and ME model, the transfer acid aspartate is decarboxylated in BSC chloroplasts by NADP-ME. The Asp+MAL and PEPCK+ME model can be regarded as an integration of these two models. In the NADP-ME model, malate is the only transfer C_4_ acid and the decarboxylase is NADP-ME. The Asp and PEPCK-only model describes a hypothetical condition that the transferred aspartate can only be decarboxylated by PEPCK. Black dots indicate experimental data from maize (Leegood and von Caemmerer, 1989). *C*_*i*_ was 150 μbar in the simulation. MAL, malate; NADP-MDH, NADPH-malate dehydrogenase; NADP-ME, NADP-malic enzyme; PEPCK, phosphoenolpyruvate carboxykinase (this figure is available in colour at *JXB* online).

The four mixed-pathway models were analysed together with a standard NADP-ME model (Fig. 1). First, the models were used to predict responses of CO_2_ assimilation rate (*A*) to the photosynthetic photon flux density (PPFD) (Fig. 2). The standard NADP-ME model had the highest predicted assimilation rate at photon flux densities below a threshold of 1000 μmol m^–2^ s^–1^. Above this threshold, a mixed model assuming transfer of Asp and malate but only decarboxylation through NADP-ME (Asp+MAL and ME) showed the highest assimilation rate. Compared to the standard NADP-ME model, all the mixed models assuming PEPCK activity had lower assimilation rates at photon flux densities below 2000 μmol m^–2^ s^–1^. Apart from the PEPCK-only model, which has no photosynthesis rate, photosynthetic responses predicted by all other models were comparable to experimental data from maize (Fig. 2; Leegood and von Caemmerer, 1989).

**Fig. 3.**
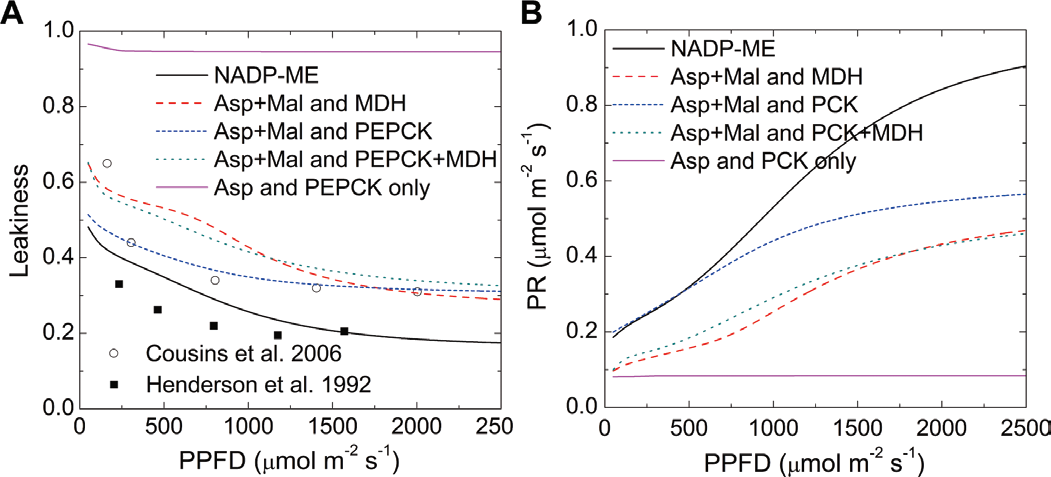
Predicted leakiness and photorespiration rate. (A) Simulated leakiness vs. photosynthetic photon flux density (PPFD) for various combinations: open circles indicate experimental data from *Flaveria bidentis* (Cousins *et al.*, 2006) and filled squared indicate experimental data from maize (Henderson *et al.*, 1992). (B) Predicted photorespiration rate vs. PPFD for various combinations. MAL, malate; NADP-MDH, NADPH-malate dehydrogenase; NADP-ME, NADP-malic enzyme; PEPCK, phosphoenolpyruvate carboxykinase (this figure is available in colour at *JXB* online).

The predicted leakiness was increased in all mixed-pathway models compared to the standard NADP-ME model (Fig. 3A). The modelled leakiness under different light levels was generally consistent with measured leakiness for maize (Fig. 3A) and *Flaveria*, two typical NADP-ME subtype C_4_ plants. The predicted photorespiratory rate was highest in the standard NADP-ME model and lower in all mixed models (Fig. 3B). The photorespiratory rate is a function of the CO_2_ and O_2_ concentration around Rubisco. In this simulation, O_2_ concentration was constant, because no LET generated O_2_ in BSCs, while there were additional pathways that increased CO_2_ concentration in BSCs (Supplementary Fig. S3 available at *JXB* online). Therefore, the simulations showed that the photorespiratory rate was suppressed in all mixed models; furthermore, compared to models including PEPCK activity, the standard NADP-ME model had a higher assimilation rate and lower leakiness while the Asp+MAL+ME model had a lower photorespiratory rate.

### Transfer metabolite gradients and concentration changes

**Fig. 4.**
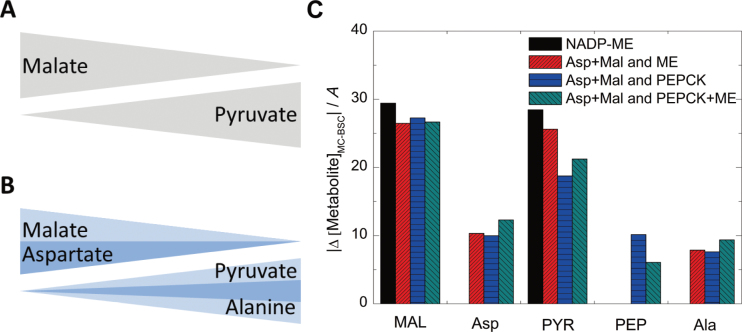
Acid gradients between mesophyll cells and bundle sheath cells. (A, B) Illustration of the gradient necessary to transfer acids between bundle sheath cells in the standard NADP-ME type (A) and mixed type (B). (C) Simulated acid gradients of different C_4_ pathway combinations (μM) normalized by CO_2_ assimilation rate (μmol m^–2^ s^–1^). The photosynthetic photon flux density used in the simulation was 2000 μmol m^–2^ s^–1^, and *C*_*i*_ was 150 μbar. MAL, malate; NADP-MDH, NADPH-malate dehydrogenase; NADP-ME, NADP-malic enzyme; PEP, phosphoenolpyruvate; PEPCK, phosphoenolpyruvate carboxykinase; PYR, pyruvate (this figure is available in colour at *JXB* online).

The mixed model presented for maize (Pick *et al.*, 2011) includes five transfer acids (Asp, malate, Ala, pyruvate, and PEP) and two decarboxylation enzymes. Yet the model predictions for assimilation rate, leakiness, and photorespiration dismissed a mixed model, because a mixed model showed lower energy-use efficiency and higher leakiness, although it suppressed photorespiration rate (Figs 2 and 3). Hence, this work tested whether a mixed model altered other parameters which may explain why the mixed pathway was realized during evolution. In theory, the concurrent use of several species of transfer acids will reduce the necessary concentration gradients for each of them (Fig. 4A and B). Movement of these metabolites between MCs and BSCs was assumed to follow a diffusional process through plasmodesmata, as has been suggested earlier (Stitt and Heldt, 1985). The transport rates are dependent on the concentration gradient and the diffusion coefficient of each individual metabolite.

The model simulation indicated that the malate gradient between MCs and BSCs was only slightly lower in all mixed models as compared to the standard NADP-ME model. The pyruvate gradient was also only slightly lower in the Asp+MAL and ME model, but it was approximately halved in the mixed model, which allowed for PEPCK activity. The reduction in pyruvate transport was compensated by concurrent transport of PEP and alanine, indicating that the model predictions confirmed the theoretical estimations with regard to the C_3_ but only marginally so for the C_4_ acids (Fig. 4C).

**Fig. 5.**
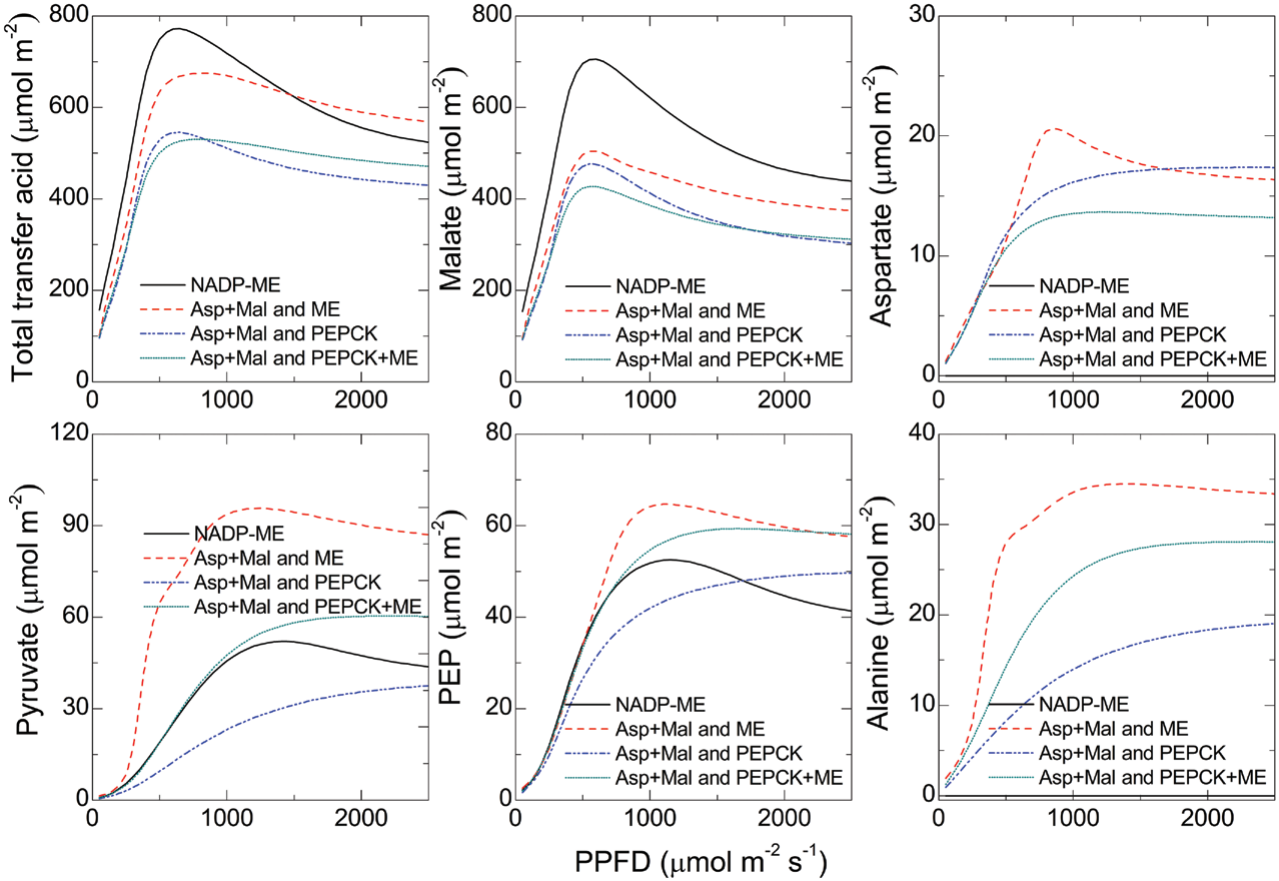
Predicted changes in amounts of transfer acids in leaf tissue with changes in photosynthetic photon flux density (PPFD). *C*_*i*_ in the simulation was 150 μbar. MAL, malate; NADP-MDH, NADPH-malate dehydrogenase; NADP-ME, NADP-malic enzyme; PEPCK, phosphoenolpyruvate carboxykinase (this figure is available in colour at *JXB* online).

The concentrations of transfer acids were also different between the models. The highest amount of transfer acids was present in the standard NADP-ME model, closely followed by the Asp+MAL and ME model. The models using PEPCK activity had lower total transfer acid contents (Fig. 5A). For malate, the standard NADP-ME model predicted the highest content, followed by the Asp+MAL and ME model; the models using PEPCK had lower malate contents (Fig. 5B). For all other transfer acids, the Asp+MAL and ME model predicted the highest contents (Fig. 5C–F), for pyruvate up to 3-times higher compared to the mixed models (Fig. 5D). It is notable that the malate contents were an order of magnitude higher compared to the other transfer acids (Fig. 5B).

In summary, the models including PEPCK activity had lower transfer acids contents and lower gradients, which matched the aforementioned theoretical considerations (Fig. 5).

### Mixed pathways shift the optimal energy allocation between BSCs and MCs and show that linear electron transport in BSCs is permissible

**Fig. 6.**
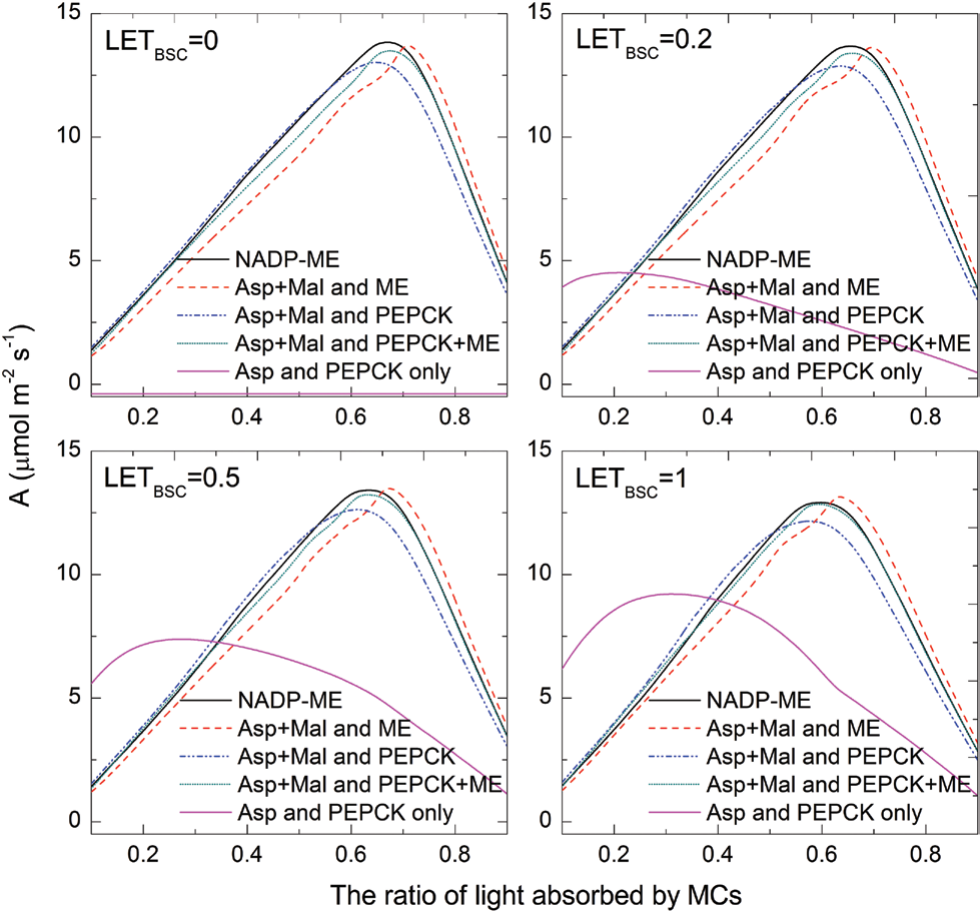
The effects of light allocation between mesophyll cells (MCs) and bundle sheath cells on photosynthetic CO_2_-uptake rate. LET_BSC_ represents the proportion of liner electron transport of the total electron transport capacity in bundle sheath cells. Photosynthetic photon flux density in the simulation was 300 μmol m^–2^ s^–1^. MAL, malate; NADP-MDH, NADPH-malate dehydrogenase; NADP-ME, NADP-malic enzyme; PEPCK, phosphoenolpyruvate carboxykinase (this figure is available in colour at *JXB* online).

Theoretically, using different transfer acids should also affect the optimum of light energy allocation between MCs and BSCs, assuming that LET is present and no reducing equivalents are shuttled by metabolites. However, the model predicted very similar optimal light allocations for four different assumptions of LET (Fig. 6); hence, reducing equivalents could not exclusively result from LET but must be shuttled also by metabolites. All LET-BSC assumptions predicted that 60–70% of light is allocated to the MCs for optimal photosynthetic assimilation rates. Compared to a standalone NADP-ME type C_4_ photosynthesis, the Asp+MAL and ME model was predicted to have a higher photosynthesis rate if MCs absorbed more than 70% of incident light, while the Asp+MAL and PEPCK model was predicted to have a higher photosynthesis rate if MCs absorbed less than 50% of light (Fig. 6). The highest rates achieved mirror those of Fig. 2, with the standard model reaching the highest assimilation rate followed by the Asp+MAL and ME model, followed by the models using PEPCK.

The curves for all models except for the Asp+PEPCK model were essentially invariant in various LET ratios in BSCs. All curves peaked at similar assimilation values, indicating that different assumptions of BSC LET permitted similarly optimal photosynthetic rates.

### Photosystem II in BSCs is essential for a pathway where only aspartate and PEPCK are used

**Fig. 7.**
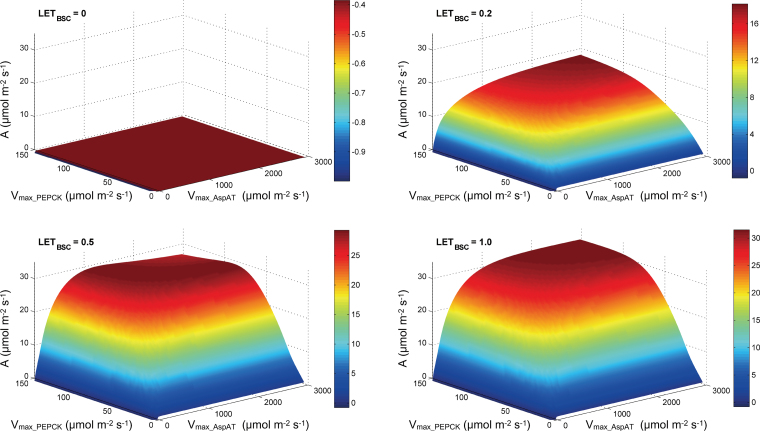
Predicted CO_2_-assimilation rate of the PEPCK-only pathway. MAL, malate; AspAT, aspartate aminotransferase; LET, linear electron transport; PEPCK, phosphoenolpyruvate carboxykinase (this figure is available in colour at *JXB* online).

When no LET was assumed in BSCs, the model which assumed only aspartate as the transfer acid and only PEPCK as the decarboxylase, predicted a zero photosynthetic CO_2_ uptake rate, no matter how high the PEPCK and AspAT activities were set (Fig. 7). Aspartate does not transfer reducing equivalents to BSCs and the latter cannot generate NADPH through light reactions in the absence of LET, therefore no NADPH to support the CO_2_ assimilation by the Calvin–Benson cycle in BSCs is available in this scenario. In a system with LET in the BSCs, the rate of photosynthetic CO_2_ uptake was proportional to the rate of LET in the BSCs. As shown in Fig. 6, the optimal energy allocation was in MCs and BSCs was 20 and 80%, respectively, of total incident light energy.

## Discussion

This work first discusses the potential physiological significance of having mixed C_4_ pathways on the basis of theoretical analyses using a systems modelling approach. Then it discusses whether the current classification of C_4_ subtypes should be reconsidered.

This work tested different models of C_4_ photosynthesis with regard to transfer acids and decarboxylation enzymes. The models indicated that a standard NADP-ME model was favoured with regard to total photosynthetic rate at low PPFD, because higher leakiness of mixed models decreases energy-use efficiency at low PPFD. Under high light, models with ME as the decarboxylation enzyme were superior over models that assumed PEPCK as the sole or partial decarboxylation enzyme (Fig. 2). A higher CO_2_ concentration in the BSC chloroplasts will increase photosynthetic efficiency of Rubisco. However, PEPCK releases CO_2_ in BSC cytosol, while NADP-ME releases CO_2_ in BSC chloroplasts, which hence shows superiority under high-light conditions (Supplementary Fig. S3 available at *JXB* online). Leakiness was also lowest for the classical NADP-ME model. Having a mixed pathway increased the overall C_4_ pathway activity since it reduced limitations on acid generation and transport in the C_4_ cycle, while the Calvin–Benson cycle activity stayed the same. The faster C_4_ cycle released more CO_2_ in BSCs (enhanced overcycling), which increased the CO_2_ concentration in BSCs (Supplementary Fig. S3 available at *JXB* online). As a result, leakiness increased in all mixed models (Fig. 3A). Additional pathways should increase leakiness, but the model might somewhat overestimate the increase in leakiness. Because of the limitation of ordinary differential equation models, the current models could not include the positioning of the organelles and therefore neglected cytosolic resistance, which is also a part of the total bundle sheath resistance, especially important for the PEPCK and NAD-ME subtype (von Caemmerer and Furbank RT, 2003). This is the most likely reason for the overestimation of leakiness in the current models, especially for leakiness of CO_2_ released by PEPCK in BS cytosol (Fig. 3A). Yet, for many C_4_ species, PEPCK activity in addition to NADP-ME or NAD-ME activity, is well documented (Meister *et al.*, 1996; Wingler *et al.*, 1999; Muhaidat *et al.*, 2007; Pick *et al.*, 2011; Sommer *et al.*, 2012; Muhaidat and McKown, 2013). Hence, this work interrogated the models to identify factors that might explain why mixed models were favoured in evolution.

### Physiological significance of having mixed C_4_ pathways

In a mixed pathway, in addition to malate and pyruvate, also aspartate, PEP, and alanine are used as transfer acids between MCs and BSCs. Utilizing two C_4_ acids and three C_3_ acids can reduce the diffusion requirements for any one acid between MCs and BSCs (Pick *et al.*, 2011), because the total flux of the C_4_ and C_3_ acids is shared between two or three metabolites. The simulations indicated that a mixed pathway reduced the metabolite concentrations and gradients required to sustain the C_4_ cycle (Fig. 4A and B).

In the model, since the flux through Asp was set to around 25%, according to the carbon label partition experiment by Hatch (1971), the gradient necessary for malate was reduced but not halved, while the gradients for pyruvate, especially for the mixed models which include PEPCK activity, were substantially reduced for each transfer acid (Fig. 4C). This is because the flux through the C_3_ acid can be shared by pyruvate, PEP, and alanine in a mixed pathway. This might at least partially explain the phenomenon that no concentration gradient was measured for pyruvate between BSCs and MCs in maize (Stitt and Heldt, 1985). Until now, no C_4_ species has been reported in which only one C_4_ and one C_3_ transfer acid is in use; hence, evolution has implemented multiple transfer acids in all C_4_ plants studied so far.

Concomitant with the decreased concentration gradients, mixed C_4_ pathways also lead to decreased concentrations of transfer acids, except the Asp+MAL and ME type under high light (Fig. 5). The finding that the Asp+MAL and ME type had higher acid concentrations has two possible explanations. One is that this model displayed a higher photosynthesis rate and higher leakiness (Figs 2 and 3), which indicates that C_4_ cycle flux in the Asp+MAL and ME type was much higher than other models: higher fluxes demand relatively higher metabolite concentrations. The other reason is that the simulated pyruvate concentration was much higher than other models’ predictions. In the Asp+MAL and ME model, although both alanine and pyruvate moved back to MCs, all of the alanine was needed to form pyruvate again before transport into MC chloroplasts to generate PEP by pyruvate phosphate dikinase. Therefore, in the Asp+MAL and ME model, more pyruvate accumulated in MCs, and the concentration of pyruvate in BSCs was also higher, to maintain the concentration gradient for diffusion. Having a decreased concentration of transfer acids might also prevent excessive accumulation of acidic substances, and decreasing the concentration of pyruvate can be beneficial because pyruvate is highly diffusible and thus its fluxes are difficult to control.

The recruitment of amino transferases to the C_4_ cycle might have been facilitated by the ubiquitous presence of relatively high activities of Asp aminotransferase and Ala aminotransferase activities in many C_3_ plants (compare with Bräutigam *et al.*, 2011; Gowik *et al.*, 2011; Sommer *et al.*, 2012). Possibly, C_4_ plants early in evolution may have initially utilized Asp as the predominant transfer acid, exploiting the fact that the presence of high amounts of glutamate as an amino donor and high amounts of OAA produced by increased PEPC activity would drive Asp aminotransferase to transaminate OAA to Asp.

In addition to reducing the concentration gradients required to run the C_4_ cycle, having a mixed pathway would increase the robustness of C_4_ metabolism by maintaining the energy balance between MCs and BSCs in response to rapidly changing light conditions. Energy balances, including both NADPH and ATP balance, in both BSCs and MCs are required for maintaining a high efficiency of C_4_ photosynthesis. This theoretical study suggested that mixed C_4_ pathways, compared to a pathway with NADP-ME subtype C_4_ photosynthesis, inherently have extra mechanisms to maintain energy balances.

First, changing the proportion of transferred C_4_ acid (i.e. the ratio of transported malate to aspartate) modulates the NADPH balance between MCs and BSCs (Evans *et al.*, 2007). If malate is used as the transfer acid, it will move NADPH to the BSCs simultaneously; while, if aspartate is used, no NADPH is moved to the BSCs. Secondly, the utilization of PEPCK, which uses ATP in BSCs, as decarboxylase, can increase the ATP requirement in BSCs. Thirdly, the PSII levels in BSCs chloroplasts control the NADPH production in BSCs, which correspondingly influences the NADPH balance. Consistent with this hypothesis, the PSII contents of BSCs in a number of species conducting NADP-ME type photosynthesis have been shown to be proportional to the amount of aspartate produced as the translocated C_4_ acid (Chapman and Hatch, 1981; Meister *et al.*, 1996). Since LET in the bundle sheath leads to the liberation of oxygen at the site of Rubisco, one may ask whether the absence of LET improves C_4_ photosynthetic efficiency. The modelling study suggests that the peak assimilation rate is independent of the amount of LET in BSCs as long as the LET ratio is half or less (Fig. 6). This may be related to why a range of BSC chloroplast morphologies, representing different amounts of LET, can be identified in C_4_ plants (Yoshimura *et al.*, 2004)

The ability to utilize multiple decarboxylases and transfer acids can potentially enable C_4_ photosynthesis to maintain higher photosynthetic efficiency under a wider range of light regimes, where allocations of light into MCs and BSCs differ. Under conditions where BSCs absorb more light, PEPCK can use the extra energy in BSCs to operate the CO_2_ pump and decrease the energy demand of pyruvate phosphate dikinase in MCs. The simulation results indicated that the Asp+MAL and PEPCK type could obtain higher photosynthesis rate if MCs absorbed less than 50% of light as compared to the NADP-ME type (Fig. 6). Under conditions where MCs absorbed more light, the usage of aspartate as the transfer acid reduced NADPH transferred from MCs to BSCs, more PGA will be moved to MCs to use the NADPH and ATP in MCs. The Asp+MAL and ME type can obtain a higher photosynthetic rate than the NADP-ME type if MC absorbs more than 70% of light (Fig. 6). Therefore, a mixed C_4_ pathway provides robustness to the pathway under fluctuating environmental conditions.

Despite having all these aforementioned benefits of mixed pathways, a mixed pathway can lead to increased leakiness, which reduces photosynthetic efficiency. However, C_4_ plants have evolved mechanisms to cope with this inevitable consequence. Modelling analysis showed that reduced PEPC activity could decrease leakiness without significantly affecting the photosynthetic rate (Supplementary Fig. S2 available at *JXB* online). If PEPC activity was decreased by 15%, leakiness was reduced by about 7% in high-light conditions. Coincidently, enzyme activity data of a leaf developmental gradient in *Z. mays* show that in the tip of the leaf PEPC activity decreased when PEPCK activity was significantly elevated (Pick *et al.*, 2011). A similar negative correlation of PEPCK and PEPC activity was also found in *C. gynandra* (Sommer *et al.*, 2012). PEPC protein amounts also slightly decreased in the tip of maize; however, the RNA expression increased in the tip (Majeran *et al.*, 2010; Pick *et al.*, 2011). The experimental evidence indicated that C_4_ plants may have evolved post-transcriptional regulatory mechanisms to limit leakiness and maintain high photosynthetic efficiency

Even with all these mechanisms to regain energy balances in BSCs and MCs, C_4_ leaves can still suffer energy imbalances between BSCs and MCs under a few artificial conditions, which correspondingly decrease photosynthetic efficiency. As an example, under blue light the photosynthetic efficiency of a C_4_ plant decreases more than that of a C_3_ plant (Evans *et al.*, 2007; Sun *et al.*, 2012). This is possibly because blue light is strongly absorbed by surface MCs (Vogelmann and Han, 2000; Vogelmann and Evans, 2002), which leaves little energy available for BSCs to gain an energy balance required to maintain a higher photosynthetic efficiency.

### C_4_ plants can be effectively classified into either NADP-ME or NAD-ME type C_4_ photosynthesis

Given these physiological significance of having mixtures of C_4_ decarboxylases and transfer acids, are there three subtypes of C_4_ photosynthesis? The standard NADP-ME model has not been realized in any C_4_ plant studied to date. *S. bicolor* might represent the Asp+MAL and ME type since no PEPCK activity has been reported. The C_4_ genus *Flaveria* also has very limited PEPCK activity (Gowik *et al.*, 2011) and thus is closer to the Asp+MAL and ME type of C_4_ photosynthesis than to the Asp+MAL and ME+PEPCK type. The Asp+MAL and ME+PEPCK model is represented by maize with NADP-ME as the ME (Pick *et al.*, 2011), by *C. gynandra* with NAD-ME as the ME (Bräutigam *et al.*, 2011; Sommer *et al.*, 2012) and by multiple other dicot species of the order *Caryophyllales* (Muhaidat and McKown, 2013). Similarly, plants that realize a PEPCK-only model have not been identified so far. The metabolic process of the PEPCK pathway usually also includes the reactions involved in the NAD-ME type C_4_ photosynthesis (Burnell and Hatch, 1988). On this aspect, plants that use PEPCK as the dominating decarboxylase but not as the only decarboxylase do exist in monocotyledons (Gutierrez *et al.*, 1974). For example, *Zoysia japonica* Steud in Eragrostoideae and *Urochloa panicoides* Beauv in Panicoideae have 3–5 times higher PEPCK activity than those of the NADP-ME and NAD-ME (Gutierrez *et al.*, 1974; Muhaidat *et al.*, 2007).

**Fig. 8.**
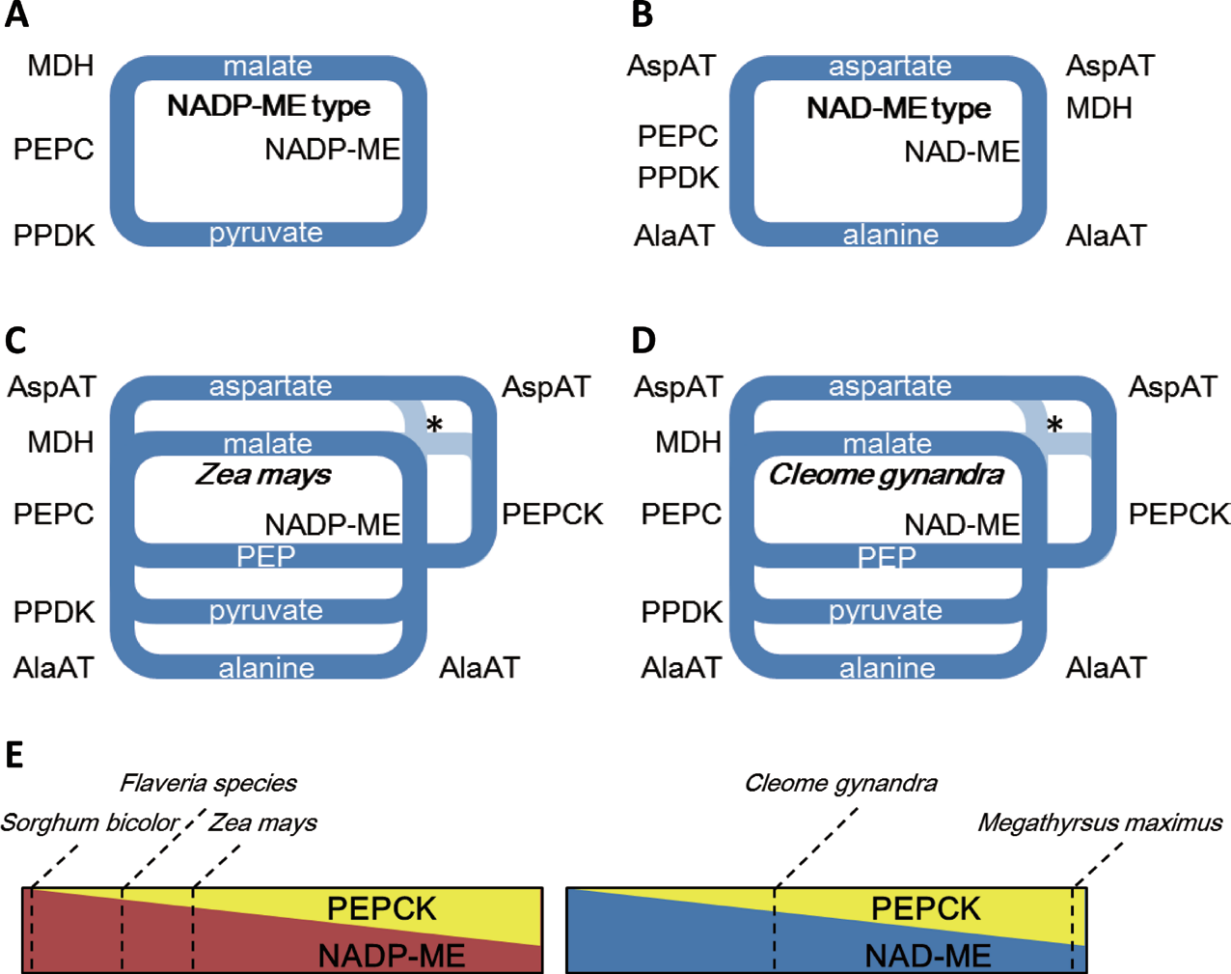
Textbook pathways (A, B) in comparison with the situation in the plant (C, D) for the NADP-ME type (A, C) and the NAD-ME type (B, D). Asterisks indicate where it is not clear whether the circles are also connected at this point by Asp aminotransferase and MDH, The C_4_ cycles have to be rewritten as branched cycles that split at the position of C_4_ transfer acid into aspartate and malate and at the position of C_3_ transfer acid into pyruvate, alanine, and (for PEPCK-using species) PEP. The proportions of different transfer acids probably vary with changing environmental conditions: for example, light for malate reduction or nitrogen availability for amino acids as transfer acids. (E) Contribution of PEPCK to malic enzyme activity in five different C_4_ species. AlaAT, alanine aminotransferase; AspAT, aspartate aminotransferase; MAL, malate; MDH, malate dehydrogenase; NADP-ME, NADP-malic enzyme; PEP, phosphoenolpyruvate; PEPC, phosphoenolpyruvate carboxylase; PEPCK, phosphoenolpyruvate carboxykinase; PPDK, pyruvate phosphate dikinase.

Recent genome-scale transcriptomic analyses in combination with enzyme activity assays have further shown that maize (Pick *et al.*, 2011) and *C. gynandra* (Sommer *et al.*, 2012) have PEPCK activity in the C_4_ cycle. Based on literature research and the modelling effort, the present work sorted these species, in addition to *Megathyrsus maximus*, a C_4_ plant classified as PEPCK type (Gutierrez *et al.*, 1974), and *S. bicolor*, a species with a relatively ‘pure’ NADP-ME type decarboxylation chemistry (Gutierrez *et al.*, 1974), according to the proportion of PEPCK in total decarboxylase activity in both NADP-ME and NAD-ME type C_4_ photosynthesis (Fig. 8E). Apparently, PEPCK plays a bigger role than previously assumed. Not only classical PEPCK plants but also NADP-ME and NAD-ME plants use substantial amounts of decarboxylation by PEPCK (see also Muhaidat *et al.*, 2007; Furbank, 2011; Pick *et al.*, 2011; Sommer *et al.*, 2012; Muhaidat and McKown, 2013). It might therefore be useful to consider C_4_ plants as either NAD-ME or NADP-ME plants, as there is a clear demarcation line based on the type of decarboxylase, with variable contributions of PEPCK (Fig. 8A–D). In other words, PEPCK might be considered as a supplementary activity to the malate-decarboxylating enzymes, not as an independent C_4_ decarboxylation activity, with the benefits of providing additional mechanisms to balance energy between BSCs and MCs and helping to decrease the metabolite concentrations of the transported acids.

However, many C_4_ dicots and monocots (e.g. sorghum; Fig. 8E), do not show high activities of PEPCK in some measurements (Gutierrez *et al.*, 1974; Muhaidat *et al.*, 2007), which possibly indicates that these species do not use PEPCK as a supplemental decarboxylation enzyme. This model analysis showed that standalone NADP-ME pathway also has good photosynthetic performance in many cases (Figs 2, 3, and 6). Even within the same plant, PEPCK activity can be flexible (e.g. high PEPCK activity is only detected in the tip of a maize leaf and in old leaves of cleome; Pick *et al.*, 2011; Sommer *et al.*, 2012). Therefore, the current analysis shows that the single NADP-ME pathway and the mixed pathways each have their own advantages (Figs 2–6): if the flux allocation in different pathways is flexible in C_4_ plants, these plants can have better adaptability under changing environmental conditions.

Can the PEPCK pathway exists in isolation: i.e. is there a C_4_ photosynthetic subtype where no NAD-ME or NADP-ME exists at all? Analysis from this study showed that photosystem II in BSCs is essential for a pure PEPCK C_4_ photosynthetic pathway that uses only PEPCK as the decarboxylase and only aspartate as the C_4_ transfer acid from MCs to BSCs (Fig. 7). This is because NADPH, which is required for 3-PGA reduction in the Calvin Benson Cycle, cannot be generated in such a C_4_ pathway unless there is LET in BSCs (i.e. only if there is PSII in BSCs). A higher LET rate in BSCs is needed for a high photosynthesis rate (Fig. 7) in such a pathway. In this case, PEPCK releases CO_2_ in BSCs cytosol and uses ATP in BSCs. Thus a PEPCK-only type prefers more energy allocated to BSCs. The current theoretical analysis suggests that the optimal energy allocation was 20% in MCs and 80% in BSCs. This, however, is next to impossible to realize because of the structure of the Kranz anatomy, where BSCs are surrounded by MCs and correspondingly light will be inevitably preferentially absorbed by MCs before reaching the BSCs. This unbalanced energy supply and usage in BSCs and MCs might have prevented evolution of C_4_ plants with PEPCK as the only decarboxylase and aspartate as the only transfer acid.

Another possibility that may limit the existence of the PEPCK-only pathway is the inability to maintain a proper amino group balance between BSCs and MCs. In the PEPCK pathway, aspartate is transported from MCs to BSCs, and PEP was transported from BSCs back to MCs, which creates an imbalance of amino groups between two cell types (Weber and Bräutigam, 2013). This problem can be solved in the PEPCK-only type model if amino groups are transported back to MCs in the form of alanine, and a reversed pyruvate transport together with alanine keeps both amino group and carbon transport balanced in both MCs and BSCs.

## Conclusion

In summary, this modelling analysis and literature survey shows that a mixed mode of C_4_ photosynthesis provides metabolic robustness through reduced metabolite gradients and metabolite concentrations. Operation of a mixed mode of C_4_ photosynthesis can confer higher ecological robustness due to increased tolerance to fluctuating light conditions. A pure PEPCK-type C_4_ photosynthesis is not beneficial because the energy requirements in BSCs cannot be fulfilled due to them being shaded by MCs. Hence, only the NAD-ME and NADP-ME subtypes should be considered as distinct subtypes, with the PEPCK pathway as a supplement to these.

## Supplementary material

Supplementary data are available at *JXB* online.

Supplementary Fig. S1. The structure of the mixed-pathway model of C_4_ photosynthesis.

Supplementary Fig. S2. Decreased PEPC reduced leakiness level of the Asp+MAL and PEPCK+ME model.

Supplementary Fig. S3. Simulated CO_2_ concentration of BSC cytosol and chloroplast.

Supplementary Data
