# Supplementary material for: Three distinct biochemical subtypes of C4 photosynthesis? A modelling analysis
Source: J Exp Bot. 2014 Mar 8;65(13):3567–78. doi: 10.1093/jxb/eru058 (PMC4085956; doi:10.1093/jxb/eru058)
Supplement: Supplementary Data [file supp_65_13_3567__index.html]

Three distinct biochemical subtypes of C4 photosynthesis? A modelling analysis — Three distinct biochemical subtypes of C4 photosynthesis? A modelling analysis — Supplementary Data 

# Three distinct biochemical subtypes of C4 photosynthesis? A modelling analysis

## Supplementary Data

Data files

**Files in this Data Supplement:**

- Supplementary Data - Supplementary Data
