# Supplementary material for: Three distinct biochemical subtypes of C4 photosynthesis? A modelling analysis
Source: J Exp Bot. 2014 Mar 8;65(13):3567–78. doi: 10.1093/jxb/eru058 (PMC4085956; doi:10.1093/jxb/eru058)
Supplement: Supplementary Data [file supp_eru058_jexbot114017_file001.pdf]

Supplemental Data

TABLE OF CONTENTS

1. LIST OF ABBREVIATIONS AND THEIR DEFINITIONS 2

1.1 METABOLITES 2
1.2 ENZYMES AND NUMBERING 3
1.3 METABOLITE TRANSPORT PROCESS 6

2 EQUATIONS 7

2.1 RATE EQUATIONS 7
2.1.1 Additional C4 cycle reactions 7
2.1.2 Light reactions 8
2.1.3 Metabolite transport reactions 9
2.1.4 CO_2 diffusion from air space to mesophyll cell 12
2.1.5 C4 cycle reactions 12
2.1.6 Calvin Cycle reactions 13
2.1.7 Starch synthesis reactions 15
2.1.8 Sucrose synthesis reactions 16
2.1.9 PGA Sink reaction 17
2.1.10 PGA<->PEP reaction 17
2.2. DIFFERENTIAL EQUATIONS 18
2.2.1. Metabolite concentration changes in mesophyll cell cytosol 18
2.2.2. Metabolite concentration changes in mesophyll cell chloroplast 19
2.2.3. Metabolite concentration changes in bundle sheath cell cytosol 20
2.2.4. Metabolite concentration changes in bundle sheath cell chloroplast 21
2.2.5. Metabolite concentration changes in bundle sheath cell peroxisome 22
2.3 CONSTANTS 23
2.3.1 Constants in mesophyll cell cytosol 23
2.3.2 Constants in mesophyll cell chloroplast 24
2.3.3 Conatants in bundle sheath cell cytosol 24
2.3.4 Conatants in bundle sheath cell chloroplast 24

3 PARAMETERS 26

3.3 VMAX OF PHOTOSYNTHETIC ENZYMES 26
3.4 MICHAELIS-CONSTANT AND OTHER PAREMETERS 28

4. SUPPLEMENTAL FIGURES 40

4.1. FIGURE S1 40

4.2. FIGURE S2 41

4.3. FIGURE S3 41

5 REFERENCE 42

1. List of Abbreviations and Their Definitions

1.1 Metabolites

Abbreviation	Full Name	Units
ADPG	ADP-glucose	mM
Ala	Alanine	mM
Asp	Aspartate	mM
CO_2	Carbon dioxide	mM
CA	Total adenylate nucleotide in the chloroplast stroma including ATP and ADP	mM
CN	Total of NADP+ and NADPH in chloroplast stroma	mM
CP	The total concentration of phosphate in chloroplast stroma	mM
DHAP	Dihydroxyacetone-phosphate	mM
DPGA	1,3-bisphosphoglycerate	mM
E4P	Erythrose 4-phosphate	mM
F26BP	Fructose 2,6–bisphosphate	mM
F6P	Fructose 6-phosphate	mM
FBP	Fructose 1,6–bisphosphate	mM
G1P	Glucose 1-phosphate	mM
G6P	Glucose 6-phosphate	mM
GAP	Glyceraldehyde 3-phosphate	mM
GCA	Glycollate	mM
GCEA	Glycerate	mM
Glu	Glutamate	mM
GLY	Glycine	mM
GOA	Glyoxylate	mM
HCO_3	Bicarbonate	mM
HexP	Hexose phosphate, includes F6P, G6P, and G1P	mM
HPR	Hydroxypyruvate	mM

KG	Ketoglutarate	mM
Mal	Malate	mM
O_2	Oxygen	mM
OAA	Oxaloacetate	mM
2OG	2-Oxoglutarate	mM
PEP	phosphoenolpyruvate	mM
PenP	Pentose phosphate including Ri5P, Ru5P, Xu5P	mM
PGA	3-Phosphoglycerate	mM
PGCA	3-Phosphoglycollate	mM
Pi	phosphate	mM
PPi	Pyrophosphate	mM
PYR	Pyruvate	mM
Ri5P	Ribose 5-phosphate	mM
Ru5P	Ribulose 5-phosphate	mM
RuBP	Ribulose 1,5-biphosphate	mM
S7P	Sedoheptulose 7-phosphate	mM
SBP	Sedoheptulose 1,7-bisphosphate	mM
SER	Serine	mM
SUC	Sucrose	mM
SUCP	Sucrose phosphate	mM
T3P	Triose phosphate including DHAP and GAP	mM
UDP	Uridine Diphosphate	mM
UDPG	Uridine Diphosphate Glucose	mM
UTP	Uridine Triphosphate	mM
Xu5P	Xylulose 5-phosphate	mM

1.2 Enzymes and Numbering

EC (or Model	Abbreviation	Full Name	Numbering

Defined)			in the Model
2.6.1.1M<br>2.6.1.1B	AspAT	aspartate transaminase	PCK1<br>PCK2
4.1.1.49	PEPCK	phosphoenolpyruvate carboxykinase (ATP)	PCK3
2.6.1.2B<br>2.6.1.2M	AlaTA	alanine transaminase	PCK4<br>PCK5
1.1.1.82B	MDH	Malate dehydrogenase (NADP+)	PCK6
4.2.1.1	CA	Carbonic anhydrase	1
4.1.1.31	PEPC	Phosphoenolpyruvate carboxylase	2
2.7.9.1	PPDK	Pyruvate, phosphate dikinase	5
1.1.1.82	MDH	Malate dehydrogenase (NADP+)	3
1.1.1.40	ME	NADP-Malic enzyme	4
4.1.1.39	Rubisco	Ribulose-bisphosphate carboxylase	6
			Pr1
2.7.2.3	PGAK	Phosphoglycerate kinase	7
2.7.2.3M			7Mchl
1.2.1.13	GAPDH	Glyceraldehyde-3-phosphate	8
1.2.1.13M		dehydrogenase (NADP+)	8Mchl
5.3.1.1	T3PI	Triose-phosphate isomerase	9
4.1.2.13F	Aldolase	Fructose-bisphosphate aldolase	10
4.1.2.13S			12
4.1.2.13M			Suc1
3.1.3.37	SBPase	Sedoheptulose-bisphosphatase	13
3.1.3.11	FBPase	Fructose-bisphosphatase	11
3.1.3.11M			Suc2
2.2.1.1X	Transketolase	Transketolase	14
2.2.1.1R			15
5.3.1.6	Ri5PI	Ribose-5-phosphate isomerase	16
5.1.3.1	Ru5PE	Ribulose-phosphate 3-epimerase	17
2.7.1.19	PRK	Phosphoribulokinase	18

5.3.1.9	G6PI	Glucose-6-phosphate isomerase	Sta1
5.3.1.9M			Suc5
5.4.2.2	PGM	Phosphoglucomutase	Sta2
5.4.2.2M			Suc6
2.7.7.27	GPA	Glucose-1-phosphate adenylyltransferase	Sta3
3.6.1.1	Diphosphatase	inorganic diphosphatase	Sta4
2.4.1.21	Starch synthase	Starch synthase	Sta5
2.7.1.105M	PFK	6-phosphofructo-2-kinase	Suc3
3.1.3.46M	F26BPP	Fructose-2,6-bisphosphate 2-phosphatase	Suc4
2.7.7.9M	UGPU	UTP-glucose-1-phosphate uridylyltransferase	Suc7
2.4.1.14M	SPS	Sucrose-phosphate synthase	Suc8
3.1.3.24M	SPP	Sucrose-phosphate phosphatase	Suc9
3.1.3.18	PGCAP	Phosphoglycolate phosphatase	Pr2
1.1.3.15	GO	(S)-2-hydroxy-acid oxidase & Catalase(CAT, EC1.11.1.6)	Pr3
2.6.1.4	GGAT	Glycine transaminase	Pr4
2.6.1.45	SGAT	Serine-glyoxylate transaminase	Pr6
1.1.1.29	HPR	Glycerate dehydrogenase	Pr7
2.7.1.31	GLYK	Glycerate kinase	Pr8
3.6.1.14M	ATPase	ATP synthase	ATPM
3.6.1.14B			ATPB
1.18.1.2M	FNR	Ferredoxin-NADP+ reductase	NADPHM
PGASink	PGASink	PGA used for amino acid synthesis or other metabolic pathway	PGASink
Mutase& Enolase	Ex	5.4.2.1&4.2.1.11	Ex

Gly_ser	Gly_Ser	EC 1.4.4.2&EC2.1.2.1	Pr5
StarchDag	StarchDag	Starch degradation	StaDag

1.3 Metabolite Transport Through Chloroplast Membrane

**Model Defined Numbering**	**Abbreviation**	**Full Name**
$\mathbf{T_{OAAB}}$	T_{OAAB} (DiT2?)	Dicarboxylate transporter
$\mathbf{T_{PGAM}}$	TPT M	Triose phosphate translocator in mesophyll cell, which can transport PGA DHAP and GAP.
$\mathbf{T_{DHAPM}}$		
$\mathbf{T_{GAPM}}$		
$\mathbf{T_{PGAM}}$	TPT B	Triose phosphate translocator in bundle sheath cell
$\mathbf{T_{DHAPM}}$		
$\mathbf{T_{GAPM}}$		
$\mathbf{T_{OAAM}}$	DiT1	Dicarboxylate transporter
$\mathbf{T_{MALM}}$		
$\mathbf{T_{MALB}}$	DiT2	
$\mathbf{T_{PEPM}}$	PPT	PEP/phosphate translocator
$\mathbf{T_{PYRM}}$	MEPM	proton:pyruvate cotransporter

2 Equations

2.1 Rate Equations

2.1.1 Additional C4 cycle reactions

$$v_{PCK1} = \frac{V_{m\_PCK1} \cdot \left([OAA]_{MC} \cdot [Glu]_{MC} - \frac{[OG]_{MC} \cdot [Asp]_{MC}}{k_{e\_PCK1}} \right)}{K_{mOAA\_PCK1} \cdot K_{mGlu\_PCK1} \cdot \alpha_{PCK1}}$$

$$\alpha_{PCK1} = 1 + \frac{[OAA]_{MC}}{K_{mOAA\_PCK1}} + \frac{[Glu]_{MC}}{K_{mGlu\_PCK1}} + \frac{[OG]_{MC}}{K_{mOG\_PCK1}} + \frac{[Asp]_{MC}}{K_{mAsp\_PCK1}} + \frac{[OAA]_{MC} \cdot [Glu]_{MC}}{K_{mOAA\_PCK1} \cdot K_{mGlu\_PCK1}} + \frac{[OG]_{MC} \cdot [Asp]_{MC}}{K_{mOG\_PCK1} \cdot K_{mAsp\_PCK1}}$$

$$v_{PCK2} = \frac{V_{m\_PCK2} \cdot \left([OG]_{BSC} \cdot [Asp]_{BSC} - \frac{[OAA]_{BSC} \cdot [Glu]_{BSC}}{k_{e\_PCK2}} \right)}{K_{mOG\_PCK2} \cdot K_{mAsp\_PCK2} \cdot \alpha_{PCK2}}$$

$$\alpha_{PCK2} = 1 + \frac{[OG]_{BSC}}{K_{mOG\_PCK2}} + \frac{[Asp]_{BSC}}{K_{mAsp\_PCK2}} + \frac{[OAA]_{BSC}}{K_{mOAA\_PCK2}} + \frac{[Glu]_{BSC}}{K_{mGlu\_PCK2}} + \frac{[OG]_{BSC} \cdot [Asp]_{BSC}}{K_{mOG\_PCK2} \cdot K_{mAsp\_PCK2}} + \frac{[OAA]_{BSC} \cdot [Glu]_{BSC}}{K_{mOAA\_PCK2} \cdot K_{mGlu\_PCK2}}$$

$$v_{PCK3} = \frac{V_{m\_PCK3} \cdot [OAA]_{BSC} \cdot [ATP]_{BSC}}{(K_{mOAA\_PCK3} + [OAA]_{BSC}) \cdot (K_{mATP\_PCK3} + [ATP]_{BSC})}$$

$$v_{PCK4} = \frac{V_{m\_PCK4} \cdot \left([Glu]_{BSC} \cdot [PYR]_{BSC} - \frac{[Ala]_{BSC} \cdot [OG]_{BSC}}{k_{e\_PCK4}} \right)}{K_{mGlu\_PCK4} \cdot K_{mPYR\_PCK4} \cdot \alpha_{PCK4}}$$

$$\alpha_{PCK4} = 1 + \frac{[Glu]_{BSC}}{K_{mGlu\_PCK4}} + \frac{[PYR]_{BSC}}{K_{mPYR\_PCK4}} + \frac{[Ala]_{BSC}}{K_{mAla\_PCK4}} + \frac{[OG]_{BSC}}{K_{mOG\_PCK4}} + \frac{[Glu]_{BSC} \cdot [PYR]_{BSC}}{K_{mGlu\_PCK4} \cdot K_{mPYR\_PCK4}} + \frac{[Ala]_{BSC} \cdot [OG]_{BSC}}{K_{mAla\_PCK4} \cdot K_{mOG\_PCK4}}$$

$$v_{PCK5} = \frac{V_{m\_PCK5} \cdot \left([Ala]_{BSC} \cdot [OG]_{BSC} - \frac{[Glu]_{BSC} \cdot [PYR]_{BSC}}{k_{e\_PCK5}} \right)}{K_{mAla\_PCK5} \cdot K_{mOG\_PCK5} \cdot \alpha_{PCK5}}$$

$$\alpha_{PCK5} = 1 + \frac{[Ala]_{BSC}}{K_{mAla\_PCK5}} + \frac{[OG]_{BSC}}{K_{mOG\_PCK5}} + \frac{[Glu]_{BSC}}{K_{mGlu\_PCK5}} + \frac{[PYR]_{BSC}}{K_{mPYR\_PCK5}} + \frac{[Ala]_{BSC} \cdot [OG]_{BSC}}{K_{mAla\_PCK5} \cdot K_{mOG\_PCK5}}$$
$$+ \frac{[Glu]_{BSC} \cdot [PYR]_{BSC}}{K_{mGlu\_PCK5} \cdot K_{mPYR\_PCK5}}$$

$$v_{PCK6} = \frac{V_{PCK6} \cdot \left([OAA]_{Bchl} \cdot [NADPH]_{Bchl} - \frac{[NADP]_{Bchl} \cdot [MAL]_{Bchl}}{k_{e\_PCK6}} \right)}{K_{mOAA\_PCK6} \cdot K_{mNADPH\_PCK6} \cdot \alpha_{PCK6}}$$

$$\alpha_{PCK6} = 1 + \frac{[OAA]_{Bchl}}{K_{mOAA\_PCK6}} + \frac{[NADPH]_{Bchl}}{K_{mNADPH\_PCK6}} + \frac{[NADP]_{Bchl}}{K_{mNADP\_PCK6}} + \frac{[MAl]_{Bchl}}{K_{mMAl\_PCK6}} + \frac{[OAA]_{Bchl} \cdot [NADPH]_{Bchl}}{K_{mOAA\_PCK6} \cdot K_{mNADPH\_PCK6}}$$
$$+ \frac{[NADP]_{Bchl} \cdot [MAl]_{Bchl}}{K_{mNADP\_PCK6} \cdot K_{mMAl\_PCK6}}$$

2.1.2 Light reactions

$$I_m = X_m \cdot I \cdot abs(1-f) \cdot \frac{1}{2}$$

$$J_{\max\_m} = Y_m \cdot J_{\max}$$

$$I_b = X_b \cdot I \cdot abs(1-f)$$

$$J_{\max\_b} = Y_b \cdot J_{\max}$$

$$J_m = \frac{I_m + J_{\max\_m} - \sqrt{\left(I_m + J_{\max\_m}\right)^2 - 4\theta I_m J_{\max\_m}}}{2\theta}$$

$$v_{ATPM} = \frac{\min(V_{m\_ATPM}, D \cdot J_m) \cdot \left([ADP]_{MC} \cdot [Pi]_{MC} - \frac{[ATP]_{MC}}{k_{e\_ATPM}} \right)}{K_{mADP\_ATPM} \cdot K_{mPi\_ATPM} \cdot \alpha_{ATPM}}$$

$$\alpha_{ATPM} = 1 + \frac{[ADP]_{MC}}{K_{mADP\_ATPM}} + \frac{[Pi]_{MC}}{K_{mPi\_ATPM}} + \frac{[ATP]_{MC}}{K_{mATP\_ATPM}} + \frac{[ADP]_{MC} \cdot [Pi]_{MC}}{K_{mADP\_ATPM} \cdot K_{mPi\_ATPM}}$$

$$v_{NADPHM} = \frac{\min(V_{m\_NADPHM}, E \cdot J_m) \cdot \left([NADP]_{Mchl} - \frac{[NADPH]_{Mchl}}{k_{e\_NADPHM}} \right)}{K_{mNADP\_NADPHM} \cdot \left(1 + \frac{[NADP]_{Mchl}}{K_{mNADP\_NADPHM}} + \frac{[NADPH]_{Mchl}}{K_{mNADPH\_NADPHM}} \right)}$$

$$v_{O_2M} = \frac{1}{2} v_{NADPHM}$$

$$I_{l\_b} = \frac{1}{2} u I_b$$

$$J_{\max\_l\_b} = v J_{\max\_b}$$

$$J_{l\_b} = \frac{I_{l\_b} + J_{\max\_l\_b} - \sqrt{\left(I_{l\_b} + J_{\max\_l\_b}\right)^2 - 4\theta I_{l\_b} J_{\max\_l\_b}}}{2\theta}$$

$$I_{c\_b} = (1-u) I_b$$

$$J_{\max\_c\_b} = (1-v) J_{\max\_b}$$

$$J_{c\_b} = \frac{I_{c\_b} + J_{\max\_c\_b} - \sqrt{\left(I_{c\_b} + J_{\max\_c\_b}\right)^2 - 4\theta I_{c\_b} J_{\max\_c\_b}}}{2\theta}$$

$$v_{ATPB} = \frac{\min(V_{m\_ATPB}, G \cdot J_{c\_b} + D \cdot J_{l\_b}) \cdot \left([ADP]_{BSC} \cdot [Pi]_{BSC} - \frac{[ATP]_{BSC}}{k_{e\_ATPB}} \right)}{K_{mADP\_ATPB} \cdot K_{mPi\_ATPB} \cdot \alpha_{ATPB}}$$

$$\alpha_{ATPB} = 1 + \frac{[ADP]_{BSC}}{K_{mADP\_ATPB}} + \frac{[Pi]_{BSC}}{K_{mPi\_ATPB}} + \frac{[ATP]_{BSC}}{K_{mATP\_ATPB}} + \frac{[ADP]_{BSC} \cdot [Pi]_{BSC}}{K_{mADP\_ATPB} \cdot K_{mPi\_ATPB}}$$

$$v_{NADPHB} = \frac{\min(V_{m\_NADPHB}, E \cdot J_{l\_b}) \cdot \left([NADP]_{Bchl} - \frac{[NADPH]_{Bchl}}{k_{e\_NADPHB}} \right)}{K_{mNADP\_NADPHB} \cdot \left(1 + \frac{[NADP]_{Bchl}}{K_{mNADP\_NADPHB}} + \frac{[NADPH]_{Bchl}}{K_{mNADPH\_NADPHB}} \right)}$$

$$v_{O_2B} = \frac{1}{2} v_{NADPHB}$$

2.1.3 Metabolite transport reactions

$$\mathrm{v}_{tAsp} = J_{Asp\_PD} \cdot \frac{S_{PD}}{S_l} = \frac{D_{Asp\_PD}}{l_{PD}} \cdot \frac{S_w \cdot \phi}{S_l} \cdot \left([Asp]_{MC} - [Asp]_{BSC} \right)$$

$$\mathrm{v}_{tAla} = J_{Ala\_PD} \cdot \frac{S_{PD}}{S_l} = \frac{D_{Ala\_PD}}{l_{PD}} \cdot \frac{S_w \cdot \phi}{S_l} \cdot \left([Ala]_{BSC} - [Ala]_{MC} \right)$$

$$\mathrm{v}_{tPEP} = J_{PEP\_PD} \cdot \frac{S_{PD}}{S_l} = \frac{D_{PEP\_PD}}{l_{PD}} \cdot \frac{S_w \cdot \phi}{S_l} \cdot \left([PEP]_{BSC} - [PEP]_{MC}\right)$$

$$\mathrm{v}_{TMAL} = J_{MAL\_PD} \cdot \frac{S_{PD}}{S_l} = \frac{D_{MAL\_PD}}{l_{PD}} \cdot \frac{S_w \cdot \phi}{S_l} \cdot \left([MAL]_{MC} - [MAL]_{BSC}\right)$$

$$\mathrm{v}_{TPYR} = J_{PYR\_PD} \cdot \frac{S_{PD}}{S_l} = \frac{D_{PYR\_PD}}{l_{PD}} \cdot \frac{S_w \cdot \phi}{S_l} \cdot \left([PYR]_{BSC} - [PYR]_{MC}\right)$$

$$\mathrm{v}_{TPGA} = J_{PGA\_PD} \cdot \frac{S_{PD}}{S_l} = \frac{D_{PGA\_PD}}{l_{PD}} \cdot \frac{S_w \cdot \phi}{S_l} \cdot \left([PGA]_{BSC} - [PGA]_{MC}\right)$$

$$\mathrm{v}_{TGAP} = J_{GAP\_PD} \cdot \frac{S_{PD}}{S_l} = \frac{D_{GAP\_PD}}{l_{PD}} \cdot \frac{S_w \cdot \phi}{S_l} \cdot \left([GAP]_{MC} - [PGA]_{BSC}\right)$$

$$\mathrm{v}_{TDHAP} = J_{DHAP\_PD} \cdot \frac{S_{PD}}{S_l} = \frac{D_{DHAP\_PD}}{l_{PD}} \cdot \frac{S_w \cdot \phi}{S_l} \cdot \left([DHAP]_{MC} - [DHAP]_{BSC}\right)$$

$$\mathrm{v}_{\mathrm{leak}} = \mathrm{v}_{tCO_2} = J_{CO_2\_PD} \cdot \frac{S_{PD}}{S_l} = \frac{D_{CO_2\_PD}}{l_{PD}} \cdot \frac{S_w \cdot \phi}{S_l} \cdot \left([CO_2]_{BSC} - [CO_2]_{MC}\right)$$

$$\mathrm{v}_{tO_2} = J_{O_2\_PD} \cdot \frac{S_{PD}}{S_l} = \frac{D_{O_2\_PD}}{l_{PD}} \cdot \frac{S_w \cdot \phi}{S_l} \cdot \left([O_2]_{BSC} - [O_2]_{MC}\right)$$

$$v_{tOAA\_B} = V_{m\_tOAA\_B} \cdot ([OAA]_{BSC} - [OAA]_{Bchl})$$

$$v_{TOAA\_M} = V_{m\_OAA\_M} \cdot \frac{\left([OAA]_{MC} - \frac{[OAA]_{Mchl}}{K_{i\_OAA\_M}}\right)}{[OAA]_{MC} + K_{m\_OAA\_M} \cdot \left(1 + \frac{[malate]_{MC}}{K_{imal\_OAA\_M}}\right)}$$

$$v_{TMAL\_M} = \frac{V_{MAL\_M} \cdot \left([MAL]_{Mchl} - [MAL]_{MC}\right)}{[MAL]_{Mchl} + K_{mMAL\_MAL\_M} \cdot (1 + \frac{[OAA]_{Mchl}}{K_{iOAA\_MAL\_M}})}$$

$$v_{TMAL\_B} = \frac{V_{MAL\_B} \cdot \left([MAL]_{BSC} - [MAL]_{Bchl}\right)}{[MAL]_{BSC} + K_{mMAL\_MAL\_B}}$$

$$v_{TPYR\_B} = \frac{V_{m\_PYR\_B} \cdot [PYR]_{Bchl}}{\left([PYR]_{Bchl} + K_{m\_PYR\_B}\right)}$$

$$v_{TPYR\_M} = \frac{V_{m\_PYR\_M} \cdot [PYR]_{MC}}{[PYR]_{MC} + K_{m\_PYR\_M}}$$

$$v_{TPEP\_M} = \frac{V_{m\_PEP\_M} \cdot [PEP]_{Mchl}}{[PEP]_{Mchl} + K_{m\_PEP\_M}}$$

$$v_{TPGA\_B} = \frac{V_{m\_C3P\_B} \cdot [PGA]_{Bchl}}{[PGA]_{Bchl} + K_{mPGA} \cdot \left(1 + \frac{[DHAP]_{Bchl}}{K_{mDHAP}}\right)\left(1 + \frac{[GAP]_{Bchl}}{K_{mGAP}}\right)} - \\ - \frac{V_{m\_C3P\_B} \cdot [PGA]_{BSC}}{[PGA]_{BSC} + K_{mPGA} \cdot \left(1 + \frac{[DHAP]_{BSC}}{K_{mDHAP}}\right)\left(1 + \frac{[GAP]_{BSC}}{K_{mGAP}}\right)}$$

$$v_{TGAP\_B} = \frac{V_{m\_C3P\_B} \cdot [GAP]_{BSC}}{[GAP]_{BSC} + K_{mGAP} \cdot \left(1 + \frac{[DHAP]_{BSC}}{K_{mDHAP}}\right)\left(1 + \frac{[PGA]_{BSC}}{K_{mPGA}}\right)} - \\ - \frac{V_{m\_C3P\_B} \cdot [GAP]_{Bchl}}{[GAP]_{Bchl} + K_{mGAP} \cdot \left(1 + \frac{[DHAP]_{Bchl}}{K_{mDHAP}}\right)\left(1 + \frac{[PGA]_{Bchl}}{K_{mPGA}}\right)}$$

$$v_{TDHAP\_B} = \frac{V_{m\_C3P\_B} \cdot [DHAP]_{BSC}}{[DHAP]_{BSC} + K_{mDHAP} \cdot \left(1 + \frac{[GAP]_{BSC}}{K_{mGAP}}\right)\left(1 + \frac{[PGA]_{BSC}}{K_{mPGA}}\right)} - \\ - \frac{V_{m\_C3P\_B} \cdot [DHAP]_{Bchl}}{[DHAP]_{Bchl} + K_{mDHAP} \cdot \left(1 + \frac{[GAP]_{Bchl}}{K_{mGAP}}\right)\left(1 + \frac{[PGA]_{Bchl}}{K_{mPGA}}\right)}$$

$$v_{TPGA\_M} = \frac{V_{m\_C3P\_B} \cdot [PGA]_{MC}}{[PGA]_{MC} + K_{mPGA} \cdot \left(1 + \frac{[DHAP]_{MC}}{K_{mDHAP}}\right)\left(1 + \frac{[GAP]_{MC}}{K_{mGAP}}\right)} - \\ - \frac{V_{m\_C3P\_B} \cdot [PGA]_{Mchl}}{[PGA]_{Mchl} + K_{mPGA} \cdot \left(1 + \frac{[DHAP]_{Mchl}}{K_{mDHAP}}\right)\left(1 + \frac{[GAP]_{Mchl}}{K_{mGAP}}\right)}$$

$$v_{TGAP\_M} = \frac{V_{m\_C3P\_B} \cdot [GAP]_{Mchl}}{[GAP]_{Mchl} + K_{mGAP} \cdot \left(1 + \frac{[DHAP]_{Mchl}}{K_{mDHAP}}\right)\left(1 + \frac{[PGA]_{Mchl}}{K_{mPGA}}\right)} - \\ - \frac{V_{m\_C3P\_B} \cdot [GAP]_{MC}}{[GAP]_{MC} + K_{mGAP} \cdot \left(1 + \frac{[DHAP]_{MC}}{K_{mDHAP}}\right)\left(1 + \frac{[PGA]_{MC}}{K_{mPGA}}\right)}$$

$$v_{TDHAP\_M} = \frac{V_{m\_C3P\_B} \cdot [DHAP]_{Mchl}}{[DHAP]_{Mchl} + K_{mDHAP} \cdot \left(1 + \frac{[GAP]_{Mchl}}{K_{mGAP}}\right)\left(1 + \frac{[PGA]_{Mchl}}{K_{mPGA}}\right)} -$$

$$- \frac{V_{m\_C3P\_B} \cdot [DHAP]_{MC}}{[DHAP]_{MC} + K_{mDHAP} \cdot \left(1 + \frac{[GAP]_{MC}}{K_{mGAP}}\right)\left(1 + \frac{[PGA]_{MC}}{K_{mPGA}}\right)}$$

$$v_{leak\_Bchl} = v_{tCO_2\_Bchl} = J_{CO_2\_Bchl} \cdot \frac{S_{chl}}{S_l} = P_{CO_2\_Bchl} \cdot \frac{S_{chl}}{S_l} \cdot \left([CO_2]_{Bchl} - [CO_2]_{BSC}\right)$$

$$v_{tO_2\_Bchl} = J_{O_2\_Bchl} \cdot \frac{S_{chl}}{S_l} = P_{O_2\_Bchl} \cdot \frac{S_{chl}}{S_l} \cdot \left([O_2]_{Bchl} - [O_2]_{BSC}\right)$$

2.1.4 CO_2 diffusion from air space to mesophyll cell

$$v_{inf} = g_m \cdot \frac{1}{S_c} \cdot 10^{-3} \cdot (C_i - [CO_2]_{MC})$$

2.1.5 C4 cycle reactions

$$v_1 = \frac{V_{m\_1} \cdot \left([CO_2]_{MC} - \frac{[HCO_3^-]_{MC} \cdot [H^+]_{MC}}{k_{e\_1}}\right)}{K_{mCO_2\_1} \cdot \left(1 + \frac{[CO_2]_{MC}}{K_{mCO_2\_1}} + \frac{[HCO_3^-]_{MC}}{K_{mHCO_3\_1}}\right)}$$

$$v_2 = \frac{V_{m\_2} \cdot [HCO_3^-]_{MC} \cdot [PEP]_{MC}}{\left([PEP]_{MC} + K'_{mPEP\_2}\right)\left([HCO_3^-]_{MC} + K_{mHCO_3\_2}\right)}$$

$$K'_{mPEP\_2} = \frac{K_{mPEP\_2} \cdot \left(1 + \frac{[MAL]_{MC}}{K_{iMAl\_2}}\right)}{\left(1 + \frac{[G6P]_{MC}}{K_{aG6P\_2}} + \frac{[T3P]_{MC}}{K_{aT3P\_2}}\right)}$$

$$v_3 = \frac{V_{m\_3} \cdot \left([OAA]_{Mchl} \cdot [NADPH]_{Mchl} - \frac{[NADP]_{Mchl} \cdot [MAL]_{Mchl}}{k_{e\_3}}\right)}{K_{mOAA\_3} \cdot K_{mNADPH\_3} \cdot \alpha_3}$$

$$\alpha_3 = 1 + \frac{[OAA]_{Mchl}}{K_{mOAA\_3}} + \frac{[NADPH]_{Mchl}}{K_{mNADPH\_3}} + \frac{[NADP]_{Mchl}}{K_{mNADP\_3}} + \frac{[MAl]_{Mchl}}{K_{mMAl\_3}} +$$

$$+ \frac{[OAA]_{Mchl} \cdot [NADPH]_{Mchl}}{K_{mOAA\_3} \cdot K_{mNADPH\_3}} + \frac{[NADP]_{Mchl} \cdot [MAl]_{Mchl}}{K_{mNADP\_3} \cdot K_{mMAl\_3}}$$

$$v_4 = \frac{V_{m\_4} \cdot \left([MAl]_{Bchl} \cdot [NADP]_{Bchl} - \frac{[PYR]_{Bchl} \cdot [NADPH]_{Bchl} \cdot [CO_2]_{Bchl}}{k_{e\_4}} \right)}{K_{mMAL\_4} \cdot K_{mNADP\_4} \cdot \alpha_4}$$

$$\alpha_4 = 1 + \frac{[MAl]_{Bchl}}{K_{mMAL\_4}} + \frac{[NADP]_{Bchl}}{K_{mNADP\_4}} + \frac{[PYR]_{Bchl}}{K_{mPYR\_4}} + \frac{[NADPH]_{Bchl}}{K_{mNADPH\_4}} + \frac{[CO_2]_{Bchl}}{K_{mCO_2\_4}} +$$
$$+ \frac{[MAl]_{Bchl} \cdot [NADP]_{Bchl}}{K_{mMAL\_4} \cdot K_{mNADP\_4}} + \frac{[PYR]_{Bchl} \cdot [NADPH]_{Bchl}}{K_{mPYR\_4} \cdot K_{mNADPH\_4}} + \frac{[PYR]_{Bchl} \cdot [CO_2]_{Bchl}}{K_{mPYR\_4} \cdot K_{mCO_2\_4}} +$$
$$+ \frac{[CO_2]_{Bchl} \cdot [NADPH]_{Bchl}}{K_{mCO_2\_4} \cdot K_{mNADPH\_4}} + \frac{[PYR]_{Bchl} \cdot [NADPH]_{Bchl} \cdot [CO_2]_{Bchl}}{K_{mPYR\_4} \cdot K_{mNADPH\_4} \cdot K_{mCO_2\_4}}$$

$$v_5 = \frac{V_{m\_5} \cdot [PYR]_{Mchl} \cdot [ATP]_{Mchl}}{\left([PYR]_{Mchl} + K_{mPYR\_5} \cdot \left(1 + \frac{[PEP]_{Mchl}}{K_{iPEP\_5}} \right) \right) \left([ATP]_{Mchl} + K_{mATP\_5} \right)}$$

2.1.6 Calvin Cycle reactions

$$\upsilon = \frac{V_{m\_6} \cdot \min\left(1, \frac{[RuBP]_{Bchl}}{[Rubisco]_{Bchl}} \right) \cdot [RuBP]_{Bchl} \cdot [CO_2]_{Bchl}}{\left([CO_2]_{Bchl} + K'_{mCO_2\_6} \right) \left([RuBP]_{Bchl} + K'_{mRuBP\_6} \right)}$$

$$K'_{mCO_2\_6} = K_{mCO_2\_6} \cdot \left(1 + \frac{[O_2]_{Bchl}}{K_{iO_2\_6}} \right)$$

$$K'_{mRuBP\_6} = K_{mRuBP\_6} \cdot \left(1 + \frac{[PGA]_{Bchl}}{K_{iPGA\_6}} + \frac{[FBP]_{Bchl}}{K_{iFBP\_6}} + \frac{[SBP]_{Bchl}}{K_{iSBP\_6}} + \frac{[Pi]_{Bchl}}{K_{iPi\_6}} + \frac{[NADPH]_{Bchl}}{K_{iNADPH\_6}} \right)$$

$$v_7 = \frac{V_{m\_7} \cdot [PGA]_{Bchl} \cdot [ATP]_{Bchl}}{\left([PGA]_{Bchl} + K_{mPGA\_7} \right) \left([ATP]_{Bchl} + K_{mATP\_7} \cdot (1 + \frac{[ADP]_{Bchl}}{K_{iADP\_7}}) \right)}$$

$$v_8 = \frac{V_{m\_8} \cdot [DPGA]_{Bchl} \cdot [NADPH]_{Bchl}}{\left([DPGA]_{Bchl} + K_{mPGA\_8} \right) \left([NADPH]_{Bchl} + K_{mNADPH\_8} \right)}$$

$$v_{10} = \frac{V_{m\_10} \cdot \left([GAP]_{Bchl} \cdot [DHAP]_{Bchl} - \frac{[FBP]_{Bchl}}{k_{e\_10}} \right)}{K_{mGAP\_10} \cdot K_{mDHAP\_10} \cdot \alpha_{10}}$$

$$\alpha_{10} = 1 + \frac{[GAP]_{Bchl}}{K_{mGAP\_10}} + \frac{[DHAP]_{Bchl}}{K_{mDHAP\_10}} + \frac{[FBP]_{Bchl}}{K_{mFBP\_10}} + \frac{[GAP]_{Bchl} \cdot [DHAP]_{Bchl}}{K_{mGAP\_10} \cdot K_{mDHAP\_10}}$$

$$v_{11} = \frac{V_{m\_11} \cdot \left([FBP]_{Bchl} - \frac{[F6P]_{Bchl} \cdot [Pi]_{Bchl}}{k_{e\_11}} \right)}{\left([FBP]_{Bchl} + K_{mFBP\_11} \cdot \left(1 + \frac{[F6P]_{Bchl}}{K_{iF6P\_11}} + \frac{[Pi]_{Bchl}}{K_{iPi\_11}} \right) \right)}$$

$$v_{12} = \frac{V_{m\_12} \cdot \left([DHAP]_{Bchl} \cdot [E4P]_{Bchl} - \frac{[SBP]_{Bchl}}{k_{e\_12}} \right)}{\left([DHAP]_{Bchl} + K_{mDHAP\_12} \right) \left([E4P]_{Bchl} + K_{mE4P\_12} \right)}$$

$$v_{13} = \frac{V_{m\_13} \cdot \left([SBP]_{Bchl} - \frac{[Pi]_{Bchl} \cdot [S7P]_{Bchl}}{k_{e\_13}} \right)}{\left([SBP]_{Bchl} + K_{mSBP\_13} \cdot \left(1 + \frac{[Pi]_{Bchl}}{K_{iPi\_13}} \right) \right)}$$

$$v_{14} = \frac{V_{m\_14} \cdot \left([F6P]_{Bchl} \cdot [GAP]_{Bchl} - \frac{[Xu5P]_{Bchl} \cdot [E4P]_{Bchl}}{k_{e\_14}} \right)}{\left([F6P]_{Bchl} + K'_{mF6P\_14} \right) \left([GAP]_{Bchl} + K_{mGAP\_14} \right)}$$

$$K'_{mF6P\_14} = K_{mF6P\_14} \cdot \left(1 + \frac{[Xu5P]_{Bchl}}{K_{iXu5P\_14}} + \frac{[E4P]_{Bchl}}{K_{iE4P\_14}} \right)$$

$$v_{15} = \frac{V_{m\_15} \cdot \left([GAP]_{Bchl} \cdot [S7P]_{Bchl} - \frac{[Ri5P]_{Bchl} \cdot [Xu5P]_{Bchl}}{k_{e\_15}} \right)}{\left([GAP]_{Bchl} + K'_{mGAP\_15} \right) \left([S7P]_{Bchl} + K_{mS7P\_15} \right)}$$

$$K'_{mGAP\_15} = K_{mGAP\_15} \cdot \left(1 + \frac{[Xu5P]_{Bchl}}{K_{iXu5P\_14}} + \frac{[Ri5P]_{Bchl}}{K_{iRi5P\_15}} \right)$$

$$v_{18} = \frac{V_{m\_18} \cdot \left([ATP]_{Bchl} \cdot [Ru5P]_{Bchl} - \frac{[ADP]_{Bchl} \cdot [RuBP]_{Bchl}}{k_{e\_18}} \right)}{\left([ATP]_{Bchl} + K'_{mATP\_18} \right)\left([Ru5P]_{Bchl} + K'_{mRu5P\_18} \right)}$$

$$K'_{mATP\_18} = K_{mATP\_18} \cdot \left(1 + \frac{[ADP]_{Bchl}}{K_{iADP\_18}} \right)$$

$$K'_{mRu5P\_18} = K_{mRu5P\_18} \cdot \left(1 + \frac{[PGA]_{Bchl}}{K_{iPGA\_18}} + \frac{[RuBP]_{Bchl}}{K_{iRuBP\_18}} + \frac{[Pi]_{Bchl}}{K_{iPi\_18}} \right)$$

$$v_{7M} = \frac{V_{m\_7M} \cdot \left([PGA]_{Mchl} \cdot [ATP]_{Mchl} \right)}{\left([PGA]_{Mchl} + K_{mPGA\_7M} \right)\left([ATP]_{Mchl} + K_{mATP\_7M} \cdot \left(1 + \frac{[ADP]_{Mchl}}{K_{iADP\_7M}} \right) \right)}$$

$$v_{8M} = \frac{V_{m\_8M} \cdot [DPGA]_{Mchl} \cdot [NADPH]_{Mchl}}{\left([DPGA]_{Mchl} + K_{mPGA\_8M} \right)\left([NADPH]_{Mchl} + K_{mNADPH\_8M} \right)}$$

2.1.7 Starch synthesis reactions

$$v_{Sta3} = \frac{V'_{m\_Sta3} \cdot \left([G1P]_{Bchl} \cdot [ATP]_{Bchl} - \frac{[ADPG]_{Bchl} \cdot [PPi]_{Bchl}}{K_{e\_Sta3}} \right)}{K_{mG1P\_Sta3} \cdot K'_{mATP\_Sta3} \cdot \alpha_{Sta3}}$$

$$V'_{m\_Sta3} = V_{m\_Sta3} \cdot \frac{[PGA]_{Bchl}}{\left([PGA]_{Bchl} + K_{aPGA\_Sta3} \right)}$$

$$K'_{mATP\_Sta3} = K_{mATP\_Sta3} \cdot \left(1 + \frac{[ADP]_{Bchl}}{K_{iAADP\_ATP\_Sta3}} + \frac{[PPi]_{Bchl}}{K_{iCPPi\_ATP\_Sta3}} + \frac{[Pi]_{Bchl}}{K_{iAPi\_ATP\_Sta3}} \right)$$

$$\alpha_{\text{Sta3}} = 1 + \frac{[G1P]_{Bchl}}{K_{mG1P\_Sta3}} + \frac{[ATP]_{Bchl}}{K'_{mATP\_Sta3}} + \frac{[ADPG]_{Bchl}}{K_{mADPG\_Sta3}} + \frac{[PPi]_{Bchl}}{K_{mPP1\_Sta3}} + \\ + \frac{[G1P]_{Bchl} \cdot [ATP]_{Bchl}}{\left(K_{mG1P\_Sta3} \cdot K'_{mATP\_Sta3} \right)} + \frac{[ADPG]_{Bchl} \cdot [PPi]_{Bchl}}{\left(K_{mADPG\_Sta3} \cdot K_{mPP1\_Sta3} \right)}$$

$$v_{Sta4} = \frac{V_{m\_Sta4} \cdot \left([PPi]_{Bchl} - \frac{[PPi]^2_{Bchl}}{K_{e\_Sta4}} \right)}{[PPi]_{Bchl} + K_{mPPi\_Sta4}}$$

$$v_{Sta5} = \frac{V_{m\_Sta5} \cdot [ADPG]_{Bchl}}{\left([ADPG]_{Bchl} + K_{mADPG\_Sta5} \right)}$$

2.1.8 Sucrose synthesis reactions

$$v_{Suc1} = \frac{V_{m\_Suc1} \cdot \left([GAP]_{MC} \cdot [DHAP]_{MC} - \frac{[FBP]_{MC}}{k_{e\_Suc1}} \right)}{K_{mGAP\_Suc1} \cdot K_{mDHAP\_Suc1} \cdot \alpha_{Suc1}}$$

$$\alpha_{Suc1} = 1 + \frac{[GAP]_{MC}}{K_{mGAP\_Suc1}} + \frac{[DHAP]_{MC}}{K_{mDHAP\_Suc1}} + \frac{[FBP]_{MC}}{K_{mFBP\_Suc1}} + \frac{[GAP]_{MC} \cdot [DHAP]_{MC}}{K_{mGAP\_Suc1} \cdot K_{mDHAP\_Suc1}}$$

$$v_{Suc2} = \frac{V_{m\_Suc2} \cdot \left([FBP]_{MC} - \frac{[F6P]_{MC} \cdot [Pi]_{MC}}{k_{e\_Suc2}} \right)}{K'_{mFBP\_Suc2} \cdot \alpha_{Suc2}}$$

$$\alpha_{Suc2} = 1 + \frac{[FBP]_{MC}}{K'_{mFBP\_Suc2}} + \frac{[F6P]_{MC}}{K_{mF6P\_Suc2}} + \frac{[Pi]_{MC}}{K_{mPi\_Suc2}} + \frac{[F6P]_{MC} \cdot [Pi]_{MC}}{K_{mF6P\_Suc2} \cdot K_{mPi\_Suc2}}$$

$$K'_{mFBP\_Suc2} = K_{mFBP\_Suc2} \cdot \left(1 + \frac{[F26BP]_{MC}}{K_{iF26BP\_Suc2}} \right)$$

$$v_{Suc3} = \frac{V_{m\_Suc3} \cdot \left([ATP]_{MC} \cdot [F6P]_{MC} - \frac{[ADP]_{MC} \cdot [F26BP]_{MC}}{k_{e\_Suc3}} \right)}{\left([ATP]_{MC} + K'_{mATP\_Suc3} \right) \left([F6P]_{MC} + K'_{mF6P\_Suc3} \right)}$$

$$K'_{mATP\_Suc3} = K_{mATP\_Suc3} \cdot \left(1 + \frac{[ADP]_{MC}}{K_{iADP\_Suc3}} \right)$$

$$K'_{mF6P\_Suc3} = K_{mF6P\_Suc3} \cdot \left(1 + \frac{[F26BP]_{MC}}{K_{iF26BP\_Suc3}} \right) \cdot \left(1 + \frac{[DHAP]_{MC}}{K_{iDHAP\_Suc3}} \right)$$

$$v_{Suc4} = \frac{V_{m\_Suc4} \cdot [F26BP]_{MC}}{K_{mF26BP\_Suc4} \cdot \left(1 + \frac{[F26BP]_{MC}}{K_{mF26BP\_Suc4}} \right) \left(1 + \frac{[Pi]_{MC}}{K_{mPi\_Suc4}} \right) \left(1 + \frac{[F6P]_{MC}}{K_{mF6P\_Suc4}} \right)}$$

$$v_{Suc7} = \frac{V_{m\_Suc7} \cdot \left([UTP]_{MC} \cdot [G1P]_{MC} - \frac{[UDPG]_{MC} \cdot [PPi]_{MC}}{k_{e\_Suc7}} \right)}{K_{mUTP\_Suc7} \cdot K_{mG1P\_Suc7} \cdot \alpha_{Suc7}}$$

$$\alpha_{Suc7} = 1 + \frac{[UTP]_{MC}}{K_{mUTP\_Suc7}} + \frac{[G1P]_{MC}}{K_{mG1P\_Suc7}} + \frac{[UDPG]_{MC}}{K_{mUDPG\_Suc7}} + \frac{[PPi]_{MC}}{K_{mPPi\_Suc7}} + \\ + \frac{[UTP]_{MC} \cdot [G1P]_{MC}}{K_{mUTP\_Suc2} \cdot K_{mG1P\_Suc2}} + \frac{[UDPG]_{MC} \cdot [PPi]_{MC}}{K_{mUDPG\_Suc2} \cdot K_{mPPi\_Suc2}}$$

$$v_{Suc8} = \frac{V_{m\_Suc8} \cdot \left([F6P]_{MC} \cdot [UDPG]_{MC} - \frac{[SUCP]_{MC} \cdot [UDP]_{MC}}{k_{e\_Suc8}} \right)}{\left([F6P]_{MC} + K'_{mF6P\_Suc8} \right) \left([UDPG]_{MC} + K'_{mUDPG\_Suc8} \right)}$$

$$K'_{mF6P\_Suc8} = K_{mF6P\_Suc8} \cdot \left(1 + \frac{[FBP]_{MC}}{K_{iFBP\_Suc8}} \right)$$

$$K'_{mUDPG\_Suc8} = K_{mUDPG\_Suc8} \cdot \left(1 + \frac{[UDP]_{MC}}{K_{iUDP\_Suc8}} \right) \left(1 + \frac{[SUCP]_{MC}}{K_{iSUCP\_Suc8}} \right) \left(1 + \frac{[SUC]_{MC}}{K_{iSUC\_Suc8}} \right) \left(1 + \frac{[Pi]_{MC}}{K_{iPi\_Suc8}} \right)$$

$$v_{Suc9} = \frac{V_{m\_Suc9} \cdot \left([SUCP]_{MC} - \frac{[SUC]_{MC} \cdot [Pi]_{MC}}{k_{e\_Suc9}} \right)}{[SUCP]_{MC} + K_{mSUCP\_Suc9} \cdot \left(1 + \frac{[SUC]_{MC}}{K_{mSUC\_Suc9}} \right)}$$

$$v_{Suc10} = V_{m\_Suc10} \cdot \frac{[SUC]_{MC}}{\left([SUC]_{MC} + K_{mSUC\_Suc10} \right)}$$

2.1.9 PGA Sink reaction

$$v_{PGAsink} = V_{m\_PGAsink} \cdot \frac{[PGA]_{MC}}{\left([PGA]_{MC} + K_{mPGA\_PGAsink} \right)}$$

2.1.10 PGA<->PEP reaction

$$v_{Ex} = \frac{V_{m\_Ex} \cdot \left([PGA]_{MC} - \frac{[PEP]_{MC}}{k_{e\_Ex}} \right)}{K_{mPGA\_Ex} \cdot \left(1 + \frac{[PGA]_{MC}}{K_{mPGA\_Ex}} + \frac{[PEP]_{MC}}{K_{mPEP\_Ex}} \right)}$$

2.2. Differential Equations

2.2.1. Metabolite concentration changes in mesophyll cell cytosol

$$\frac{d[O_2]_{MC}}{dt} = 0$$

$$\frac{d[OAA]_{MC}}{dt} = \left(v_2 - v_{tOAA\_M} - v_{PCK1} \right) \cdot \frac{1}{Vol_{Mcyto}}$$

$$\frac{d[PYR]_{MC}}{dt} = \left(v_{tPYR} - v_{tPYR\_M} + v_{PCK5} \right) \cdot \frac{1}{Vol_{Mcyto}}$$

$$\frac{d[PEP]_{MC}}{dt} = \left(v_{tPEP\_M} - v_2 + v_{Ex} + v_{tPEP} \right) \cdot \frac{1}{Vol_{Mcyto}}$$

$$\frac{d[Glu]_{MC}}{dt} = \left(v_{PCK5} - v_{PCK1} \right) \cdot \frac{1}{Vol_{Mcyto}}$$

$$\frac{d[OG]_{MC}}{dt} = \left(v_{PCK1} - v_{PCK5} \right) \cdot \frac{1}{Vol_{Mcyto}}$$

$$\frac{d[Asp]_{MC}}{dt} = \left(v_{PCK1} - v_{tAsp} \right) \cdot \frac{1}{Vol_{Mcyto}}$$

$$\frac{d[Ala]_{MC}}{dt} = \left(v_{tAla} - v_{PCK5} \right) \cdot \frac{1}{Vol_{Mcyto}}$$

$$\frac{d[CO_2]_{MC}}{dt} = \left(v_{\inf} - v_1 + v_{leak} + R_m \right) \cdot \frac{1}{Vol_{Mcyto}}$$

$$\frac{d[HCO_3]_{MC}}{dt} = \left(v_1 - v_2 \right) \cdot \frac{1}{Vol_{Mcyto}}$$

$$\frac{d[MAL]_{MC}}{dt} = \left(v_{MAL\_M} - v_{MAL} \right) \cdot \frac{1}{Vol_{Mcyto}}$$

$$\frac{d[PGA]_{MC}}{dt} = \left(v_{PGA} - v_{PGA\_M} - v_{Ex} - v_{PGASink}\right) \cdot \frac{1}{Vol_{Mcyto}}$$

$$\frac{d[FBP]_{MC}}{dt} = \left(v_{Suc1} - v_{Suc2}\right) \cdot \frac{1}{Vol_{Mcyto}}$$

$$\frac{d[UDPG]_{MC}}{dt} = \left(v_{Suc7} - v_{Suc8}\right) \cdot \frac{1}{Vol_{Mcyto}}$$

$$\frac{d[SUCP]_{MC}}{dt} = \left(v_{Suc8} - v_{Suc9}\right) \cdot \frac{1}{Vol_{Mcyto}}$$

$$\frac{d[SUC]_{MC}}{dt} = \left(v_{Suc9} - v_{Suc10}\right) \cdot \frac{1}{Vol_{Mcyto}}$$

$$\frac{d[F26BP]_{MC}}{dt} = \left(v_{Suc3} - v_{Suc4}\right) \cdot \frac{1}{Vol_{Mcyto}}$$

$$\frac{d[ATP]_{MC}}{dt} = \left(v_{tATP\_M} - v_{Suc7} - v_{Suc3}\right) \cdot \frac{1}{Vol_{Mcyto}}$$

$$\frac{d[T3P]_{MC}}{dt} = \left(v_{GAP\_M} + v_{DHAP\_M} - v_{GAP} - v_{DHAP} - 2v_{Suc1}\right) \cdot \frac{1}{Vol_{Mcyto}}$$

$$\frac{d[HexP]_{MC}}{dt} = \left(v_{Suc2} + v_{Suc4} - v_{Suc3} - v_{Suc7} - v_{Suc8}\right) \cdot \frac{1}{Vol_{Mcyto}}$$

2.2.2. Metabolite concentration changes in mesophyll cell chloroplast

$$\frac{d[O_2]_{Mchl}}{dt} = \left(v_{O_2M} - v_{tO_2\_M}\right) \cdot \frac{1}{Vol_{Mchl}}$$

$$\frac{d[OAA]_{Mchl}}{dt} = \left(v_{OAA\_M} - v_3\right) \cdot \frac{1}{Vol_{Mchl}}$$

$$\frac{d[MAL]_{Mchl}}{dt} = \left(v_3 - v_{MAL\_M}\right) \cdot \frac{1}{Vol_{Mchl}}$$

$$\frac{d[PEP]_{Mchl}}{dt} = \left(v_5 - v_{PEP\_M}\right) \cdot \frac{1}{Vol_{Mchl}}$$

$$\frac{d[PYR]_{Mchl}}{dt} = \left(v_{PYR\_M} - v_5\right) \cdot \frac{1}{Vol_{Mchl}}$$

$$\frac{d[NADPH]_{Mchl}}{dt} = \left(v_{NADGPHM} - v_3 - v_{8Mchl}\right) \cdot \frac{1}{Vol_{Mchl}}$$

$$\frac{d[ATP]_{Mchl}}{dt} = \left(v_{ATPM} - 2v_5 - v_{7Mchl} - v_{tATP}\right) \cdot \frac{1}{Vol_{Mchl}}$$

$$\frac{d[PGA]_{Mchl}}{dt} = \left(v_{PGA\_M} - v_{7Mchl}\right) \cdot \frac{1}{Vol_{Mchl}}$$

$$\frac{d[T3P]_{Mchl}}{dt} = \left(v_{8Mchl} - v_{GAP\_M} - v_{DHAP\_M}\right) \cdot \frac{1}{Vol_{Mchl}}$$

2.2.3. Metabolite concentration changes in bundle sheath cell cytosol

$$\frac{d[O_2]_{BSC}}{dt} = \left(v_{tO_2B} - v_{tO_2}\right) \cdot \frac{1}{Vol_{Bcyto}}$$

$$\frac{d[PYR]_{BSC}}{dt} = \left(v_{tPYR\_B} - v_{tPYR} - v_{PCK4}\right) \cdot \frac{1}{Vol_{Bcyto}}$$

$$\frac{d[OAA]_{BSC}}{dt} = \left(v_{PCK2} - v_{tOAA\_B} - v_{PCK3}\right) \cdot \frac{1}{Vol_{Bcyto}}$$

$$\frac{d[PEP]_{BSC}}{dt} = \left(v_{PCK3} - v_{tPEP}\right) \cdot \frac{1}{Vol_{Bcyto}}$$

$$\frac{d[ATP]_{BSC}}{dt} = \left(v_{tATP\_B} - v_{PCK3}\right) \cdot \frac{1}{Vol_{Bcyto}}$$

$$\frac{d[OG]_{BSC}}{dt} = \left(v_{PCK4} - v_{PCK2}\right) \cdot \frac{1}{Vol_{Bcyto}}$$

$$\frac{d[Glu]_{BSC}}{dt} = \left(v_{PCK2} - v_{PCK4}\right) \cdot \frac{1}{Vol_{Bcyto}}$$

$$\frac{d[Asp]_{BSC}}{dt} = \left(v_{tAsp} - v_{PCK2}\right) \cdot \frac{1}{Vol_{Bcyto}}$$

$$\frac{d[Ala]_{BSC}}{dt} = \left(v_{PCK4} - v_{tAla}\right) \cdot \frac{1}{Vol_{Bcyto}}$$

$$\frac{d[CO_2]_{BSC}}{dt} = \left(v_{Pr5} + v_{leak\_B} + v_{PCK3} + R_b - v_{leak}\right) \cdot \frac{1}{Vol_{Bcyto}}$$

$$\frac{d[T3P]_{BSC}}{dt} = \left(v_{GAP} + v_{DHAP} - v_{GAP\_B} - v_{DHAP\_B}\right) \cdot \frac{1}{Vol_{Bcyto}}$$

$$\frac{d[PGA]_{BSC}}{dt} = \left(v_{PGA\_B} - v_{PGA}\right) \cdot \frac{1}{Vol_{Bcyto}}$$

$$\frac{d[MAL]_{BSC}}{dt} = \left(v_{MAL} - v_{MAL\_B}\right) \cdot \frac{1}{Vol_{Bcyto}}$$

2.2.4. Metabolite concentration changes in bundle sheath cell chloroplast

$$\frac{d[O_2]_{Bchl}}{dt} = \left(v_{O_2B} - v_{tO_2\_B}\right) \cdot \frac{1}{Vol_{Bchl}}$$

$$\frac{d[NADPH]_{Bchl}}{dt} = \left(v_4 + v_{NADPHB} - v_7 - v_{PCK6}\right) \cdot \frac{1}{Vol_{Bchl}}$$

$$\frac{d[MAL]_{Bchl}}{dt} = \left(v_{tMAL\_B} + v_{PCK6} - v_4\right) \cdot \frac{1}{Vol_{Bchl}}$$

$$\frac{d[OAA]_{Bchl}}{dt} = \left(v_{tOAA\_B} - v_{PCK6}\right) \cdot \frac{1}{Vol_{Bchl}}$$

$$\frac{d[ATP]_{Bchl}}{dt} = \left(v_{ATPB} - v_7 - v_{18} - v_{Sta3} - v_{Pr8} - v_{tATP\_B}\right) \cdot \frac{1}{Vol_{Bchl}}$$

$$\frac{d[CO_2]_{Bchl}}{dt} = \left(v_4 - v_6 - v_{leak\_Bchl}\right) \cdot \frac{1}{Vol_{Bchl}}$$

$$\frac{d[RuBP]_{Bchl}}{dt} = \left(v_{18} - v_6 - v_{Pr1}\right) \cdot \frac{1}{Vol_{Bchl}}$$

$$\frac{d[PGA]_{Bchl}}{dt} = \left(2v_6 - v_7 - v_{PGA\_B} + v_{Pr1} + v_{Pr8}\right) \cdot \frac{1}{Vol_{Bchl}}$$

$$\frac{d[SBP]_{Bchl}}{dt} = \left(v_{12} - v_{13}\right) \cdot \frac{1}{Vol_{Bchl}}$$

$$\frac{d[S7P]_{Bchl}}{dt} = \left(v_{13} - v_{15}\right) \cdot \frac{1}{Vol_{Bchl}}$$

$$\frac{d[FBP]_{Bchl}}{dt} = \left(v_{10} - v_{11}\right) \cdot \frac{1}{Vol_{Bchl}}$$

$$\frac{d[E4P]_{Bchl}}{dt} = \left(v_{14} - v_{12}\right) \cdot \frac{1}{Vol_{Bchl}}$$

$$\frac{d[T3P]_{Bchl}}{dt} = \left(v_{GAP\_B} + v_{DHAP\_B} + v_8 - 2v_{10} - v_{14} - v_{15} - v_{12}\right) \cdot \frac{1}{Vol_{Bchl}}$$

$$\frac{d[HexP]_{Bchl}}{dt} = \left(v_{11} - v_{14} - v_{Sta3}\right) \cdot \frac{1}{Vol_{Bchl}}$$

$$\frac{d[Pent]_{Bchl}}{dt} = \left(v_{14} + 2v_{15} - v_{18}\right) \cdot \frac{1}{Vol_{Bchl}}$$

$$\frac{d[PYR]_{Bchl}}{dt} = \left(v_4 - v_{PYR\_B}\right) \cdot \frac{1}{Vol_{Bchl}}$$

$$\frac{d[PPi]_{Bchl}}{dt} = \left(v_{Sta3} - v_{Sta4}\right) \cdot \frac{1}{Vol_{Bchl}}$$

$$\frac{d[ADPG]_{Bchl}}{dt} = \left(v_{Sta3} - v_{Sta5}\right) \cdot \frac{1}{Vol_{Bchl}}$$

$$\frac{d[PGCA]_{Bchl}}{dt} = \left(v_{Pr1} - v_{Pr2}\right) \cdot \frac{1}{Vol_{Bchl}}$$

$$\frac{d[GCA]_{Bchl}}{dt} = \left(v_{Pr2} - v_{Pr9}\right) \cdot \frac{1}{Vol_{Bchl}}$$

$$\frac{d[GCEA]_{Bchl}}{dt} = \left(v_{Pr10} - v_{Pr8}\right) \cdot \frac{1}{Vol_{Bchl}}$$

2.2.5. Metabolite concentration changes in bundle sheath cell peroxisome

$$\frac{d[GCA]_{Bper}}{dt} = \left(v_{Pr9} - v_{Pr3}\right) \cdot \frac{1}{Vol_{Bper}}$$

$$\frac{d[GOA]_{Bper}}{dt} = \left(v_{Pr3} - v_{Pr4} - v_{Pr6}\right) \cdot \frac{1}{Vol_{Bper}}$$

$$\frac{d[GLY]_{Bper}}{dt} = \left(v_{Pr4} + v_{Pr6} - 2v_{Pr5}\right) \cdot \frac{1}{Vol_{Bper}}$$

$$\frac{d[SER]_{Bper}}{dt} = \left(v_{Pr5} - v_{Pr6}\right) \cdot \frac{1}{Vol_{Bper}}$$

$$\frac{d[HPR]_{Bper}}{dt} = \left(v_{Pr6} - v_{Pr7}\right) \cdot \frac{1}{Vol_{Bper}}$$

$$\frac{d[GCEA]_{Bper}}{dt} = \left(v_{Pr7} - v_{Pr10}\right) \cdot \frac{1}{Vol_{Bper}}$$

2.3 Constants

2.3.1 Constants in mesophyll cell cytosol

$$[CA]_{MC} = [ATP]_{MC} - [ADP]_{MC}$$

$$[CU]_{MC} = [UDP]_{MC} + [UTP]_{MC} + [UDPG]_{MC}$$

$$\begin{aligned}[CP]_{MC} = {} & [PiT]_{MC} + 2[FBP]_{MC} + 2[F26BP]_{MC} + [PGA]_{MC} + [T3P]_{MC} + \\ & + [HexP]_{MC} + [SUCP]_{MC} + [UTP]_{MC} + [ATP]_{MC} + [PEP]_{MC}\end{aligned}$$

$$[Pi]_{MC} = \frac{1}{2}(\sqrt{K_{ePi}{}^{2} + 4 \cdot K_{ePi} \cdot [PiT]_{MC}} - K_{ePi})$$

$$[PPi]_{MC} = [PiT]_{MC} - [Pi]_{MC}$$

$$[GAP]_{MC} = \frac{K_{e\_9} \cdot [T3P]_{MC}}{(1 + K_{e\_9})}$$

$$[DHAP]_{MC} = \frac{[T3P]_{MC}}{(1 + K_{e\_9})}$$

$$[G6P]_{MC} = \frac{[HexP]_{MC}}{\frac{1}{K_{e\_Suc5}} + K_{e\_Suc6} + 1}$$

$$[G1P]_{MC} = \frac{K_{e\_Suc6} \cdot [HexP]_{MC}}{\frac{1}{K_{e\_Suc5}} + K_{e\_Suc6} + 1}$$

$$[F6P]_{MC} = \frac{\frac{[HexP]_{MC}}{K_{e\_Suc5}}}{\frac{1}{K_{e\_Suc5}} + K_{e\_Suc6} + 1}$$

2.3.2 Constants in mesophyll cell chloroplast

$$[CA]_{Mchl} = [ATP]_{Mchl} + [ADP]_{Mchl}$$

$$[CN]_{Mchl} = [NADPH]_{Mchl} + [NADP]_{Mchl}$$

$$[CP]_{Mchl} = [Pi]_{Mchl} + [PGA]_{Mchl} + 2[DPGA]_{Mchl} + [T3P]_{Mchl} + [ATP]_{Mchl} + [PEP]_{Mchl}$$

$$[GAP]_{Mchl} = \frac{K_{e\_9} \cdot [T3P]_{Mchl}}{(1 + K_{e\_9})}$$

$$[DHAP]_{Mchl} = \frac{[T3P]_{Mchl}}{(1 + K_{e\_9})}$$

2.3.3 Conatants in bundle sheath cell cytosol

$$[GAP]_{BSC} = \frac{K_{e\_9} \cdot [T3P]_{BSC}}{(1 + K_{e\_9})}$$

$$[DHAP]_{BSC} = \frac{[T3P]_{BSC}}{(1 + K_{e\_9})}$$

2.3.4 Conatants in bundle sheath cell chloroplast

$$[CA]_{Bchl} = [ATP]_{Bchl} + [ADP]_{Bchl} + [ADPG]_{Bchl}$$

$$[CN]_{Bchl} = [NADPH]_{Bchl} + [NADP]_{Bchl}$$

$$\begin{aligned}[CP]_{Bchl} = {} & [Pi]_{Bchl} + [PGA]_{Bchl} + 2[DPGA]_{Bchl} + [T3P]_{Bchl} + 2[FBP]_{Bchl} + \\ & + [HexP]_{Bchl} + [E4P]_{Bchl} + 2[SBP]_{Bchl} + [S7P]_{Bchl} + [Pent]_{Bchl} + \\ & + 2[RuBP]_{Bchl} + [ATP]_{Bchl} + [PGCA]_{Bchl} + 2[PPi]_{Bchl}\end{aligned}$$

$$[GAP]_{Bchl} = \frac{K_{e\_9} \cdot [T3P]_{Bchl}}{(1 + K_{e\_9})}$$

$$[DHAP]_{Bchl} = \frac{[T3P]_{Bchl}}{(1 + K_{e\_9})}$$

$$[G6P]_{Bchl} = \frac{[HexP]_{Bchl}}{\frac{1}{K_{e\_Sta1}} + K_{e\_Sta2} + 1}$$

$$[G1P]_{Bchl} = \frac{K_{e\_Sta2} \cdot [HexP]_{Bchl}}{\frac{1}{K_{e\_Sta1}} + K_{e\_Sta2} + 1}$$

$$[F6P]_{Bchl} = \frac{\frac{[HexP]_{Bchl}}{K_{e\_Sta1}}}{\frac{1}{K_{e\_Sta1}} + K_{e\_Sta2} + 1}$$

$$[Xu5P]_{Bchl} = \frac{\frac{[PenP]_{Bchl}}{K_{e\_17}}}{\frac{1}{K_{e\_16}} + \frac{1}{K_{e\_17}} + 1}$$

$$[Ru5P]_{Bchl} = \frac{[PenP]_{Bchl}}{\frac{1}{K_{e\_16}} + \frac{1}{K_{e\_17}} + 1}$$

$$[Ri5P]_{Bchl} = \frac{\frac{[PenP]_{Bchl}}{K_{e\_16}}}{\frac{1}{K_{e\_16}} + \frac{1}{K_{e\_17}} + 1}$$

3 Parameters

3.3 Vmax of photosynthetic enzymes

EC	Numbering	Vmax (μmol m-2 s-1)	Reference
**2.6.1.1M**	PCK1	400	Assumed
**2.6.1.1B**	PCK2	400	Assumed
**4.1.1.49**	PCK3	60	Assumed
**2.6.1.2B**	PCK4	400	Assumed
**2.6.1.2M**	PCK5	400	Assumed
**1.1.1.82**	PCK6	60	Assumed
**4.2.1.1**	1	200000 or variable	Hatch and Burnell (1990) with modification
**4.1.1.31**	2	170 or variable	Kanai and Edwards (1999), Hatch (1987), von Caemmerer (2000) with modification
**1.1.1.82**	3	90 or variable	Kanai and Edwards (1999), Hatch (1987) with modification
**1.1.1.40**	4	90 or variable	Kanai and Edwards (1999), Hatch (1987) with modification
**2.7.9.1**	5	90 or variable	Kanai and Edwards (1999), Hatch (1987) with modification
**4.1.1.39**	6	65 or variable	Kanai and Edwards (1999), Hatch (1987), von Caemmerer (2000) with modification
**2.7.2.3 & 1.2.1.13**	7 and 8	225 or variable	Laisk and Edwards. (2000), with modification
**4.1.2.13FBP**	10	58.5 or variable	Zhu *et al.* (2007) with modification
**3.1.3.11**	11	43.6 or variable	Zhu *et al.* (2007) with modification
**4.1.2.13SBP**	12	110 or variable	Zhu *et al.* (2007) with modification
**3.1.3.37**	13	29.2 or variable	Zhu *et al.* (2007) with modification

**2.2.1.1X**	14	281 or variable	Zhu *et al.* (2007) with modification
**2.2.1.1R**	15	281 or variable	Zhu *et al.* (2007) with modification
**2.7.1.19**	18	1170 or variable	Zhu *et al.* (2007) with modification
**2.7.2.3M & 1.2.1.13M**	7M and 8M	300 or variable	Laisk and Edwards. (2000), with modification
**4.1.2.13FBPM**	Suc1	8.05 or variable	Zhu *et al.* (2007) with modification
**3.1.3.11M**	Suc2	6.40 or variable	Zhu *et al.* (2007) with modification
**2.7.7.9**	Suc7	5.77 or variable	Zhu *et al.* (2007) with modification
**2.4.1.14**	Suc8	27.8 or variable	Zhu *et al.* (2007) with modification
**3.1.3.24**	Suc9	27.8 or variable	Zhu *et al.* (2007) with modification
**2.7.1.105**	Suc3	1.01 or variable	Zhu *et al.* (2007) with modification
**3.1.3.46**	Suc4	0.841 or variable	Zhu *et al.* (2007) with modification
**2.7.7.27**	Sta3	30 or variable	Zhu *et al.* (2007) with modification
**3.6.1.1**	Sta4	1000 or variable	Zhu *et al.* (2007) with modification
**2.4.1.21**	Sta5	25 or variable	Zhu *et al.* (2007) with modification
**StarchDag**	StarchDag	1 or 0	Assumed
**PGASink**	PGASink	2 or variable	Zhu *et al.* (2007) with modification
**4.1.1.39PR**	Pr1	Vm_6*0.11 or variable	Cousins *et al.* (2010) with modification
**3.1.3.18**	Pr2	2621 or variable	Zhu *et al.* (2007) with modification
**1.1.3.15**	Pr3	72.8 or variable	Zhu *et al.* (2007) with modification
**2.6.1.4**	Pr4	137 or variable	Zhu *et al.* (2007) with modification
**Gly_ser**	Pr5	125 or variable	Zhu *et al.* (2007) with modification
**2.6.1.45**	Pr6	165 or variable	Zhu *et al.* (2007) with modification
**1.1.1.29**	Pr7	500 or variable	Zhu *et al.* (2007) with modification
**2.7.1.31**	Pr8	286 or variable	Zhu *et al.* (2007) with modification
**Tgca**	Pr9	300 or variable	Zhu *et al.* (2007) with modification
**Tgcea**	Pr10	250 or variable	Zhu *et al.* (2007) with modification
**5.4.2.1&4.2.1.11**	Ex	1 or variable	Laisk and Edwards. (2000), with modification

**JmaxM**	JmaxM	300 or variable	von Caemmerer (2000) with modification
**JmaxB**	JmaxB	JmaxM *(1-Y)/Y or variable	Assumed
**3.6.3.14M**	ATPM	300 or variable	Assumed
**3.6.3.14B**	ATPB	300 or variable	Assumed
**1.18.1.2M**	NADPHM	200 or variable	Assumed
**TPTM**	TPGAM, TDHAPM, TGAPM	750 or variable	Assumed
**TPTB**	TPGAM, TDHAPM, TGAPM	750 or variable	Assumed
**DiT**	TOAAM	80 or variable	Assumed
	TmalB	150 or variable	Assumed
	TmalM	150 or variable	Assumed
**PPT**	TPEPM	150 or variable	Assumed
**MEPM**	TpyrM	150 or variable	Assumed
**MEPB**	TpyrB	150 or variable	Assumed

3.4 Michaelis-constant and other paremeters

EC	Numbering	parameters	Reference
**2.6.1.1M**	PCK1	KmAsp_PCK1=2.6mM	Taniguchi et al. (1995)
		KmOxog_PCK1=0.14mM	Taniguchi et al. (1995)
		KmGlu_PCK1=17mM	Taniguchi et al. (1995)
		KmOAA_PCK1=0.065mM	Taniguchi et al. (1995)
		Ke_PCK1=1/0.148	Krebs (1953)
**2.6.1.1B**	PCK2	KmAsp_PCK2=2.6mM	Taniguchi et al. (1995)
		KmOxog_PCK2=0.14	Taniguchi et al. (1995)
		KmGlu_PCK2=17	Taniguchi et al. (1995)
		KmOAA_PCK2=0.065	Taniguchi et al. (1995)

		Ke_PCK2=0.148	Krebs (1953)
**4.1.1.49**	PCK3	KmOAA_PCK3=0.085	Chen et al. (2002)
		KmATP_PCK3=0.0257	
**2.6.1.2B**	PCK4	KmPyr_PCK4=0.33	Son et al. (1991)
		KmGlu_PCK4=5	Son et al. (1991)
		KmAla_PCK4=6.67	Son et al. (1991)
		KmOxog_PCK4=0.15	Son et al. (1991)
		Ke_PCK4=1	Green et al. (1945)
**2.6.1.2M**	PCK5	KmPyr_PCK5=0.33	Son et al. (1991)
		KmGlu_PCK5=5	Son et al. (1991)
		KmAla_PCK5=6.67	Son et al. (1991)
		KmOxog_PCK5=0.15	Son et al. (1991)
		Ke_PCK5=1	Green et al. (1945)
**1.1.1.82**	PCK6	KmNADPH_PCK6 =0.024	Kagawa and Bruno (1988)
		KmOAA_PCK6 =0.056	Kagawa and Bruno (1988)
		KmNADP_PCK6 =0.073	Kagawa and Bruno (1988)
		Kmmal_PCK6 =32.0	Kagawa and Bruno (1988)
		Ke_PCK6 =4450.0	Laisk and Edwards (2000)
**Inf**	Inf	g_m=0.7 mol m^{-2} bar^{-1}	Assumed
		Sc= 0.33 × 10^{-4} mmol $L^{-1}$$\mu bar^{-1}$	Uchida *et al.* (1983), Hoofd *et al.* (1986)
**4.2.1.1**	1	K_{mCO2_1}=2.8 mM	Hatch and Burnell (1990)
		K_{mHCO3_1}=34 mM	Pocker and Miksch (1978)
		[H+]=10e-7.3mM	Felle and Bertl (1986)
		K_{e_1} = 5.6e-7mM	Pocker and Miksch (1978)
**4.1.1.31**	2	K_{mHCO3_2} =0.02 mM	Uedan and Sugiyama (1976)
		K_{mPEP_2} = 0.1 mM	Mukerji (1977)
		K_{imal_2} =0. 5 mM	Gao and Woo (1996)

**1.1.1.82**	3	K_{mNADPH_3} =0.024 mM	Kagawa and Bruno (1988)
		K_{mOAA_3} =0.056 mM	Kagawa and Bruno (1988)
		K_{mNADP_3} =0.073 mM	Kagawa and Bruno (1988)
		K_{mmal_3} =32.0 mM	Kagawa and Bruno (1988)
		K_{e_3} =4450.0	Laisk and Edwards (2000)
**1.1.1.40**	4	K_{mCO2_4} =1.1 mM	Jenkins *et al.* (1987)
		K_{mNADP_4} =0.0080 mM	Detarsio *et al.* (2003)
		K_{mNADPH_4} =0.045 mM	Ziegler (1974)
		K_{mPyr_4} =3.0 mM	Ziegler (1974)
		K_{mmal_4} =0.23 mM	Detarsio *et al.* (2003)
		K_{e_4} =0.051 mM	Harary *et al.* (1953)
**2.7.9.1**	5	K_{iPEP_5} =0.15 mM	Jenkins and Hatch (1985)
		K_{mATP_5} =0.082 mM	Jenkins and Hatch (1985)
		K_{mPyr_5} =0.082 mM	Jenkins and Hatch (1985)
**4.1.1.39**	6	K_{mCO2_6} =0.0162 mM	Cousins *et al.* (2010)
		K_{mO2_6} =0.222 mM	Cousins *et al.* (2010)
		K_{mRuBP_6} =0.02 mM	Farquhar (1979)
		K_{iPGA_6} =2.52 mM	Assumed, Badger and Lorimer (1981)
		K_{iFBP_6} =0.04 mM	Badger and Lorimer (1981)
		K_{iSBP_6} =0.75 mM	Badger and Lorimer (1981)
		K_{iPi_6} =3.6 mM	Assumed, Badger and Lorimer (1981)
		K_{iNADPH_6} =0.21 mM	Assumed, Badger and Lorimer (1981)
**2.7.2.3 &**	7 and 8	K_{mPGA_78}=1 mM	Laisk and Edwards (2000)
**1.2.1.13**		K_{mATP_78}=0.3 mM	Laisk and Edwards (2000)

		K_{mNADPH_78}=0.05 mM	Ferri *et al.*(1978), Trost (1993), Macioszek and Anderson (1987), Baalmann *et al.*, 1995, Sparla *et al.*, (2004), Sparla *et al.*, (2005),
**5.3.1.1**	9	K_{e_9}=0.05	Bassham and Krause (1969)
**4.1.2.13FBP**	10	K_{mDHAP_10} =0.4 mM	Iwaki *et al.* (1991)
		K_{mGAP_10} =0.3 mM	Iwaki *et al.* (1991), Zhu *et al.* (2007)
		K_{mFBP_10} =0.02 mM	Brooks and Criddle (1966), Schnarrenberger and Kruger (1986)
		K_{e_10} =7.1 mM^{-1}	Bassham and Krause (1969), Iwaki *et al.* (1991)
**3.1.3.11**	11	K_{iF6P_11} =0.7 mM	Heldt (1983)
		K_{iPi_11} =12.0 mM	Charles and Halliwell (1981)
		K_{mFBP_11} =0.033 mM	Charles and Halliwell (1981)
		K_{e_11} =666000.0 mM	Bassham and Krause (1969) , Laisk *et al.*(1989)
**4.1.2.13SBP**	12	K_{mDHAP_12} =0.4 mM	Iwaki *et al.* (1991)
		K_{mE4P_12} =0.2 mM	Zhu *et al.* (2007)
		K_{mSBP_12}=0.02 mM	Brooks and Criddle (1966)
		K_{e_12} =1.017 mM^{-1}	Bassham and Krause (1969) , Laisk *et al.*(1989)
**3.1.3.37**	13	K_{iPi_13} =12.0 mM	Woodrow *et al.* (1983)
		K_{mSBP_13} =0.05 mM	Woodrow *et al.* (1983), Cadet and Meunier (1988)

		K_{e_13} =666000.0 mM	Bassham and Krause (1969), Laisk *et al.*(1989)
**2.2.1.1X**	14	K_{mE4P_14} =0.1 mM	Zhu *et al.* (2007)
		K_{mF6P_14} =0.1 mM	Zhu *et al.* (2007)
		K_{mGAP_14} =0.1 mM	Sprenger *et al.* (1995), Schenk *et al.* (1998), Zhu *et al.* (2007)
		K_{mXu5P} =0.1 mM	Schenk *et al.* (1998), Laisk *et al.* (1989), Zhu *et al.* (2007)
		K_{e_14} =0.084	Datta *et al.* (1961).
**2.2.1.1R**	15	K_{mGAP_15} =0.072 mM	Albe (1991); Laisk *et al.* (1989)
		K_{mRi5P_15} =1.5 mM	Albe (1991); Laisk *et al.* (1989)
		K_{mS7P_15} =0.46 mM	Albe (1991); Laisk *et al.* (1989)
		K_{mXu5P_15} =0.1 mM	Albe (1991); Laisk *et al.* (1989)
		K_{e_15} =1.176	Bassham and Krause (1969), Laisk *et al.* (1989)
**5.3.1.6**	16	K_{e_16}=0.4	Bassham and Krause (1969)
**5.1.3.1**	17	K_{e_17}=0.67	Bassham and Krause, (1969)
**2.7.1.19**	18	K_{iADP_18} =2.5 mM	Gardemann *et al.* (1983)
		$K_{i\_ADP\_18}$ =0.4 mM	Gardemann *et al.* (1983)
		K_{iPGA_18} =2.0 mM	Gardemann *et al.* (1983)
		K_{iPi_18} =4.0 mM	Gardemann *et al.* (1983)
		K_{iRuBP_18} =0.7 mM	Gardemann *et al.* (1983)
		K_{mATP_18} =0.625 mM	Slabas *et al.* (1976)
		K_{mRu5P_18} =0.05 mM	Gardemann *et al.* (1983), Omnaas *et al.*(1985)
		K_{e_18} =6846.0	Bassham and Krause (1969), Laisk *et al.* (1989)

**4.1.2.13FBPM**	Suc1	K_{mDHAP_Suc1}=0.45 mM	Iwaki *et al.* (1991)
		K_{mGAP_Suc1} =0.04 mM	Iwaki *et al.* (1991)
		K_{mFBP_Suc1}=0.023 mM	Schnarrenberger (1986)
		K_{e_Suc1} =12.0 mM^{-1}	Thomas *et al.* (1997), Zhu *et al.* (2007)
**3.1.3.11M**	Suc2	K_{iF26BP_Suc2} =0.007mM	Jang *et al.* (2003)
		K_{iF6P_Suc2} =0.7 mM	Heldt *et al.* (1983)
		K_{iPi_Suc2} =12.0 mM	Charles & Halliwell (1981)
		K_{mFBP_Suc2} =0.0025mM	Jang *et al.* (2003)
		K_{e_Suc2} =174.0 mM	Lawson *et al.* (1976)
**5.3.1.9M**	Suc5	K_{e_Suc5}=2.3	Bassham and Krause (1969),
**5.4.2.2M**	Suc6	K_{e_Suc6}=0.0584	Bassham and Krause (1969),
**2.7.7.9**	Suc7	K_{mG1P_Suc7} =0.14 mM	Nakano *et al.* (1989)
		K_{mPPi_Suc7} =0.11 mM	Nakano *et al.* (1989)
		K_{mUDPG_Suc7} =0.12mM	Nakano *et al.* (1989)
		K_{mUTP_Suc7} =0.1 mM	Nakano *et al.* (1989)
		K_{e_Suc7} =0.31 mM	Hansen *et al.* (1966).
**2.4.1.14**	Suc8	K_{iFBP_Suc8} =0.8 mM	Harbron *et al.* (1981)
		K_{iPi_Suc8} =11.0 mM	Harbron *et al.* (1981)
		K_{iSuc_Suc8} =50.0 mM	Salermo and Pontis (1978)
		K_{iSucP_Suc8} =0.4 mM	Harbron *et al.* (1981)
		K_{iUDP_Suc8} =0.7 mM	Harbron *et al.* (1981)
		K_{mF6P_Suc8} =0.8 mM	Lunn and Rees (1990)

		K_{mUDPG_Suc8} =2.4 mM	Lunn and Rees (1990)
		K_{e_Suc8} =10.0	Lunn and Rees (1990)
**3.1.3.24**	Suc9	K_{mSuc_Suc9} =80.0 mM	Cumino (2001)
		K_{mSucP_Suc9} =0.35 mM	Whitaker (1984)
		K_{e_Suc9} =780.0	Zhu *et al.* (2007)
**2.7.1.105**	Suc3	K_{iADP_Suc3} =0.16 mM	Kretschmer and Hofmann (1984)
		K_{iDHAP_Suc3} =0.7 mM	Markham and Kruger (2002)
		K_{mATP_Suc3} =0.5 mM	Walker and Huber (1987), Markham and Kruger (2002)
		K_{mF26BP_Suc3} =0.021 mM	Garcia de Frutos and Baanante (1995)
		K_{mF6P_Suc3} =0.5 mM	Walker and Huber (1987), Markham and Kruger (2002)
		K_{e_Suc3} =590.0	Cornish-Bowden (1997)
**3.1.3.46**	Suc4	K_{iF6P_Suc4} =0.1 mM	Villadsen and Nielsen (2001)
		$K_{iPi\_Suc}4$ =0.5 mM	Villadsen and Nielsen (2001)
		K_{mF26BP_Suc4}= 0.032 mM	Macdonald *et al.* (1989)
**5.3.1.9**	Sta1	K_{e_Sta1}=2.3	Bassham and Krause (1969)
**5.4.2.2**	Sta2	K_{e_Sta2}=0.058	Colowick and Sutherland (1942)
**2.7.7.27**	Sta3	K_{aPGA_Sta1}=0.2252 mM	Assumed
		K_{mG1P_Sta3}=0.038 mM	Fuchs *et al.* (1979)
		K_{mATP_Sta3}=0.12 mM	Boehlein *et al.* (2005)
		$K_{iPi\_ATP\_Sta3}$=2.96 mM	Boehlein *et al.* (2005)
		K_{mPPi_Sta3}=0.033 mM	Amir *et al.* (1972)
		$K_{iCPP1\_ATP\_Sta3}$=13.8E-4	Amir *et al.* (1972)

		mM	
		K_{mADPG_Sta3}=0.24 mM	Sowokinos (1981)
		$K_{iADP\_ATP\_Sta3}$=2.0 mM	Ghosh *et al.* (1966)
		K_{e_Sta3}=1.1	Espada (1962)
**3.6.1.1**	Sta4	K_{mPPi_Sta2}=0.154 mM	Van *et al.* (2005)
		K_{e_Sta2}=1.57 E-4 mM	Flodgaard *et al.* (1974)
**2.4.1.21**	Sta5	K_{mADPG_Sta3}=0.077 mM	Hawker *et al.* (1974)
**PGASink**	PGAsink	$K_{mPGA\_PGASink}$=2.4 mM	assumed
**4.1.1.39PR**	PR1	K_{mCO2_PR1}=0.0162 mM	Cousins *et al.* (2010)
		K_{mO2_PR1}=0.222 mM	Cousins *et al.*(2010)
		K_{mRuBP_PR1}=0.02 mM	Farquhar (1979)
		K_{iPGA_PR1}=2.52 mM	Assumed Badger and Lorimer (1981)
		K_{iFBP_PR1}=0.04 mM	Badger and Lorimer (1981)
		K_{iSBP_PR1}=0.75 mM	Badger and Lorimer (1981)
		K_{iPi_PR1}=3.6 mM	Assumed Badger and Lorimer (1981)
		K_{iNADPH_PR1}=0.21 mM	Assumed Badger and Lorimer (1981)
**3.1.3.18**	PR2	K_{mPGCA_PR2}=0.026 mM	Christeller and Tolbert (1978)
		K_{iPI_PR2}=2.55 mM	Christeller and Tolbert (1978)
		K_{iGCA_PR2}=94.0 mM	Christeller and Tolbert (1978)
**1.1.3.15**	PR3	K_{mGCA_PR3}= 0.1 mM	Tolbert (1981)
**2.6.1.4**	PR4	K_{e_PS4}= 607.0	Cooper and Meister (1972)
		K_{mGOA_PS4}=0.15 mM	Nakamura and Tolbert (1983)
		K_{mGLU_PS4}= 1.7 mM	Nakamura and Tolbert (1983)

		K_{iGLY_PS4}=2.0 mM	Zhu et al (2007)
**2.6.1.45**	PR6	K_{e_PR6}= 0.24	Guynn (1982)
		K_{mGOA_PR6}=0.15 mM	Nakamura and Tolbert (1983)
		K_{mSER_PR6}=2.7 mM	Nakamura and Tolbert (1983)
		K_{iGLY_PR6}=33.0 mM	Nakamura and Tolbert (1983)
**1.1.1.29**	PR7	K_{e_PR7}= 2.5 E-5	Guynn (1982), Zhu et al (2007)
		K_{iHPR_PR7}= 12.0 mM	Kleczkowski and Edwards (1989)
		K_{mHPR_PR7}=0.09 mM	Kleczkowski and Edwards (1989)
**2.7.1.31**	PR8	K_{e_PR8}= 300.0	Kleczkowski *et al.* (1985)
		K_{mATP_PR8}= 0.21 mM	Kleczkowski *et al.* (1985)
		K_{mGCEA_PR8}=0.25 mM	Kleczkowski *et al.* (1985)
		K_{iPGA_PR8}=0.72 mM	Assumed
**Gly_ser**	PR5	K_{mGLY_PS5}= 6.0 mM	Douce *et al.* (2001)
		K_{iSER_PS5}=4.0 mM	Douce *et al.* (2001)
**Tgca**	PR9	K_{mGCA_PR9}= 0.2 mM	Howitz and McCarty (1985)
		K_{iGCEA_PR9}= 0.22 mM	Howitz and McCarty (1985)
**Tgcea**	PR10	K_{mGCEA_PR10}= 0.39mM	Howitz and McCarty (1986)
		K_{iGCA_PR10}= 0.28 mM	Howitz and McCarty (1986)
**5.4.2.1&4.2.1.11**	Ex	K_{mPGA_62} = 0.1 mM	Laisk and Edwards (2000)
		K_{mPEP_62} = 0.5 mM	Laisk and Edwards (2000)
		Ke_62=0.4302	Laisk and Edwards (2000)

**3.6.3.14M**	ATPM	K_{mADP_ATPM} =0.014 mM	Davenport and Mccarty (1986)
		K_{mATP_ATPM} =0.11 mM	Penefsky (1974)
		K_{mPi_ATPM} =0.3 mM	Aflalo and Shavit (1983)
		K_{e_ATPM} =5.734 mM-1	Bassham and Krause (1969), Laisk *et al.*(1989)
		X =0.667	Assumed light partition coefficient
		Y =0.6	Assumed Jmax partition coefficient
		F =0.7225	von Caemmerer (2000) F=abs(1-f)
		θ=0.7	von Caemmerer (2000)
		D =1	von Caemmerer (2000) ATP/e- whole chain +Qcycle
**3.6.3.14B**	ATPB	K_{mADP_ATPB} =0.014 mM	Davenport and Mccarty (1986)
		KmPi_ATPB =0.11 mM	Penefsky (1974)
		KmATP_ATPB =0.3 mM	Aflalo and Shavit (1983)
		K_{e_ATPB} =5.734 mM^{-1}	Bassham and Krause (1969), Laisk et al.(1989)
		G =0.667	von Caemmerer (2000) ATP/e-
**1.18.1.2M**	NADPHM	K_{mNADP_NADPHM}=0.0072 mM	Shin (1972)
		K_{mNADPH_NADPHM}=0.036 mM	Gozzer *et al.* (1977)
		K_{e_NADPHM} =502	Knaff (1996), Keirns (1972), Laisk and Edwards (2000)
		E =0.5	von Caemmerer (2000)
**1.18.1.2M**	NADPHB	K_{mNADP_NADPHM}=0.0072 mM	Shin (1972)
		K_{mNADPH_NADPHM}=0.036	Gozzer *et al.* (1977)

		mM	
		K_{e_NADPHM} =502	Knaff (1996), Keirns (1972), Laisk and Edwards (2000)
		E =0.5	von Caemmerer (2000)
		u	Assumed light partition coefficient of linear electron transport in BSC
		v	Assumed Jmax partition coefficient of linear electron transport in BSC
**Metabolite transport through Plasmodesmata**	Leak	D_{CO2_PD}=1.7×10-9	Evans *et al.* (2009)
		l_{PD}=0.4μm	von Caemmerer and Furbank (2003)
		S_W/S_l=0.83	Sowinski *et al.* (2008)
		ϕ=0.03	Assumed
	TMAL	D_{MAL_PD}=6.67×10^{-10}	Sowinski et al (2008)
	TPYR	D_{PYR_PD}=7.00×10^{-10}	Sowinski et al (2008)
	TPGA	D_{PGA_PD}=5.25×10^{-10}	Sowinski et al (2008)
	TGAP	D_{GAP_PD}=5.25×10^{-10}	Sowinski et al (2008)
	TDHAP	D_{DHAP_PD}=5.25×10^{-10}	Sowinski et al (2008)
	TAsp	D_{Asp_PD}=6.40×10^{-10}	Sowinski et al (2008)
	TAla	D_{Ala_PD}=8.40×10^{-10}	Sowinski et al (2008)
	TPEP	D_{PEP_PD}=6.30×10^{-10}	Sowinski et al (2008)
	TO_2	D_{O2_PD}=1.8×10-9	Assumed, according to D_{CO2_PD}
**Leak_Bchl**	Leak_Bchl	P_{CO2_B}=0.002 cm s^{-1}	Evans et al (2009) Uehlein et al. (2008)
	TO2_Bchl	P_{O2_B}=0.002 cm s^{-1}	Assumed, according to P_{CO2_B}
		S_{Chl}/S_l =10	Assumed
**DiT1**	TOAAM	$K_{m\_OAA\_M}$=0.053	Hatch *et al.* (1984)
		$K_{imal\_OAA\_M}$=7.5	Hatch *et al.* (1984)
	TmalM	$K_{m\_MAL\_M}$=0.5	Day and Hatch (1981)
		$K_{iOAA\_MAL\_M}$=0.3	Day and Hatch (1981)

**DiT2**	TmalB	$K_{m\_MAL\_B}$=1	Assumed
**PPT**	TPEPM	$K_{m\_PEP\_M}$=0.3	Assumed
**MEPM**	TpyrM	$K_{m\_PYR\_M}$=0.05	Assumed
**MEPB**	TpyrB	$K_{m\_PYR\_B}$=0.05	Assumed
**TPTM**	TPGAM	K_{mPGA} = 2	Assumed
	TGAPM	K_{mGAP} = 2	Assumed
	TDHAPM	K_{mDHAP} = 2	Assumed
**TPTB**	TPGAB	K_{mPGA} = 2	Assumed
	TGAPB	K_{mGAP} = 2	Assumed
	TDHAPB	K_{mDHAP} = 2	Assumed

4. Supplemental Figures

4.1. Figure S1

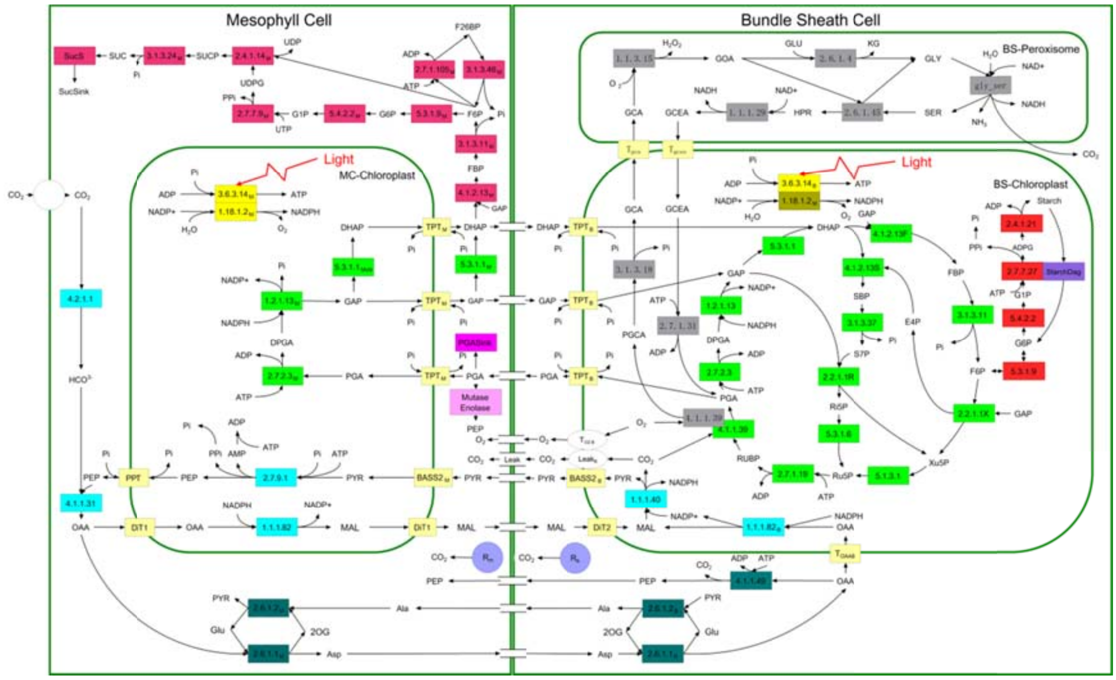

Figure S1 The structure of mixed-pathway model of C4 photosynthesis.

The rectangles indicate enzymes or transporters; colors differentiate these by function. Green: Calvin Benson cycle; Pale yellow: transporters; Grey: photorespiratory pathway; Cyan: C4 dicarboxylate cycle; Red: starch and sucrose synthesis; Yellow: light reactions; dark cyan: additional C4 cycle Enzymes are denoted by their EC numbers (Tables 2 and S1). R_m and R_b represent mitochondria respiration in MCs and BSCs

4.2. Figure S2

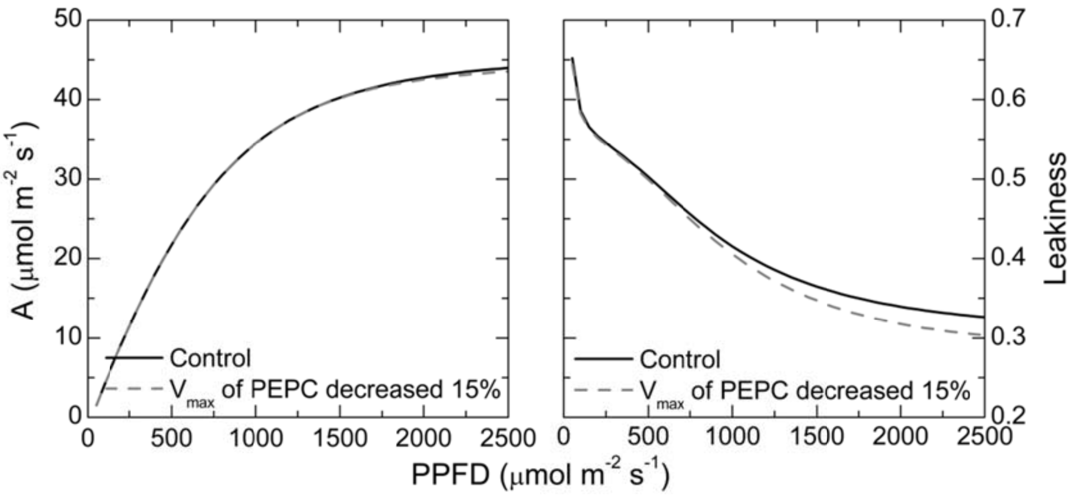

Figure S2 Decreased PEPC reduced leakiness level of 'Asp+Mal and PEPCK+ME' model

4.3. Figure S3

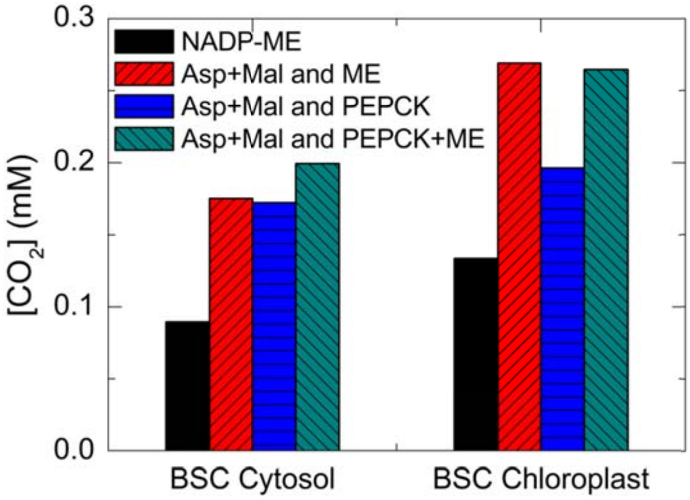

Figure S3 Simulated CO_2 concentration of BSC cytosol and chloroplast. The PPFD used in the simulation was 2000 μmol m^{-2} s^{-1}, and C_i was 150 μbar.

5 Reference

Aflalo C, Shavit N (1983) Steadystate kinetics of photophosphorylation: limited access of nucleotides to the active site on the ATP synthetase. FEBS Lett. 154: 1 75-79

Agostino A, Jeffrey P, Hatch MD (1992) Amino Acid Sequence and Molecular Weight of Native NADP Malate Dehydrogenase from the C(4) Plant Zea mays. Plant Physiol 98: 1506-1510

Albe KR (1991) Partial purification and kinetic characterization of transaldolase from Dictyostelium discoideum. Exp Mycol 15:255–62

Amir J, Cherry JH (1972) Purification and properties of adenosine diphosphoglucose pyrophosphorylase from sweet corn. Plant Physiol 49: 893-897

Baalmann E, Backhausen JE, Rak C, Vetter S, Scheibe R (1995) Reductive modification and nonreductive activation of purified spinach chloroplast NADP-dependent glyceraldehyde-3-phosphate dehydrogenase. Arch Biochem Biophys 324: 201-208

Bassham JA, Krause GH (1969). Free energy changes and metabolic regulation in steady-state photosynthetic carbon reduction. Biochim Biophys Acta 189: 207-221

Badger MR, Lorimer GH (1981) Interaction of sugar phosphates with the catalytic site of ribulose-1,5-bisphosphate carboxylase. Biochemistry-Us 20: 2219-2225

Baez M, Rodriguez PH, Babul J, Guixe V (2003) Structural and functional roles of Cys-238 and Cys-295 in Escherichia coli phosphofructokinase-2. Biochem J 376: 277-283

Bassham JA, Krause GH (1969) Free energy changes and metabolic regulation in steady-state photosynthetic carbon reduction. Biochim Biophys Acta 189: 207-221

Bentahir M, Feller G, Aittaleb M, Lamotte-Brasseur J, Himri T, Chessa JP, Gerday C (2000) Structural, kinetic, and calorimetric characterization of the cold-active phosphoglycerate kinase from the antarctic Pseudomonas sp. TACII18. J Biol Chem 275: 11147-11153

Brooks K, Criddle RS (1966) Enzymes of the carbon cycle of photosynthesis. I. Isolation and properties of spinach chloroplast aldolase. Arch Biochem Biophys 117:650–659

Boehlein SK, Sewell AK, Cross J, Stewart JD, Hannah LC (2005) Purification and characterization of adenosine diphosphate glucose pyrophosphorylase from maize/potato mosaics. Plant Physiol 138: 1552-1562

Cadet F, Meunier JC (1988) pH and kinetic studies of chloroplast sedoheptulose-1,7

-bisphosphatase from spinach (Spinacia oleracea). Biochem J 253: 249-254

Cadet F, Meunier JC, Ferte N (1987) Isolation and purification of chloroplastic spinach (Spinacia oleracea) sedoheptulose-1,7-bisphosphatase. Biochem J 241: 71-74

Charles SA, Halliwell B (1981) Light activation of fructose bisphosphatase in photosynthetically competent pea chloroplasts. Biochem J 200: 357-363

Chen ZH, Walker RP, Acheson RM, Leegood RC (2002) Phosphoenolpyruvate carboxykinase assayed at physiological concentrations of metal ions has a high affinity for CO_2 Plant Physiol. 128, 160-164

Christeller JT, Tolbert NE (1978) Phosphoglycolate phosphatase. Purification and properties. J Biol Chem 253: 1780-1785

Colowick SP, Sutherland EW (1942) Polysaccharide synthesis from glucose by means of purified enzymes. J Biol Chem 144: 423-437

Cooper JL, Meister A (1972) Isolation and properties of highly purified glutamine transaminase. Biochemistry-Us 11: 661-671

Cousins AB, Ghannoum O, Von Caemmerer S, Badger MR (2010) Simultaneous determination of Rubisco carboxylase and oxygenase kinetic parameters in Triticum aestivum and Zea mays using membrane inlet mass spectrometry. Plant Cell Environ 33: 444-452

Cumino A, Ekeroth C, Salerno GL (2001) Sucrose-phosphate phosphatase from Anabaena sp. strain PCC 7120: isolation of the protein and gene revealed significant structural differences from the higher-plant enzyme. Planta 214: 250-256

Datta AG, Racker E (1961) Mechanism of action of transketolase. I. Properties of the crystalline yeast enzyme. J Biol Chem 236: 617-623

Davenport JW, McCarty RE (1986) Relationships between rates of steady-state ATP synthesis and the magnitude of the protonactivity gradient across thylakoid membranes. Biochim Biophys Acta 851: 136–145

Day DA, Hatch MD (1981) Dicarboxylate Transport in Maize Mesophyll Chloroplasts. Arch Biochem Biophys 211: 738-742

Detarsio E, Wheeler MC, Campos Bermudez VA, Andreo CS, Drincovich MF (2003) Maize C4 NADP-malic enzyme. Expression in Escherichia coli and characterization of site-directed mutants at the putative nucleoside-binding sites. J Biol Chem 278: 13757-13764

Dietz KJ, Heber U (1984) Rate-limiting factors in leaf photosynthesis. 1. Carbon fluxes in the Calvin cycle. Biochim Biophys Acta 767: 432–443

Douce R, Bourguignon J, Neuburger M, Rebeille F (2001) The glycine decarboxylase system: a fascinating complex. Trends Plant Sci 6: 167-176

Echeverria E, Salerno G (1994) Properties of sucrose-phosphate phosphatase from rice (Oryza sativa) leaves. Plant Sci. 96, 15-19

Espada J (1962) Enzymic synthesis of adenosine diphosphate glucose from glucose-l-phosphate and adenosine triphosphate. J Biol Chem 237:3577-3581

Evans JR, Kaldenhoff R, Genty B, Terashima I (2009) Resistances along the CO2 diffusion pathway inside leaves. J Exp Bot 60: 2235-2248

Farquhar GD (1979) Models describing the kinetics of ribulose biphosphate carboxylase-oxygenase. Arch Biochem Biophys 193: 456-468

Felle H, Bertl A (1986) The Fabrication Of H+-Selective Liquid-Membrane Microelectrodes for Use In Plant-Cells. J Exp Bot 37: 1416-1428

Ferri G, Comerio G, Iadarola P, Zapponi MC, Speranza ML (1978) Subunit structure and activity of glyceraldehyde-3-phosphate dehydrogenase from spinach chloroplasts. Biochim Biophys Acta 522: 19-31

Fifis T, Scopes RK (1978) Purification of 3-phosphoglycerate kinase from diverse sources by affinity elution chromatography. Biochem J 175: 311-319

Flodgaard H, Fleron P (1974) Thermodynamic parameters for the hydrolysis of inorganic pyrophosphate at pH 7.4 as a function of (Mg2+), (K+), and ionic strength determined from equilibrium studies of the reaction. J Biol Chem 249: 3465-3474

Fuchs RL, Smith JD (1979) The purification and characterization of ADP-glucose pyrophosphorylase A from developing maize seeds. Biochim Biophys Acta 566: 40-48

Gao Y, Woo KC (1996) Regulation of phosphoenolpyruvate carboxylase in Zea mays by protein phosphorylation and metabolites and their roles in photosynthesis. Aust J Plant Physiol 23: 25-32

Garcia de Frutos P, Baanante IV (1995) The muscle isoform of 6-phosphofructo 2-kinase/fructose 2,6-bisphosphatase of the teleost Sparas aurata: relationship with the liver isoform. Arch. Biochem. Biophys. 321: 297–302

Gardemann A (1983) Control of CO2 fixation: regulation of spinach ribulose-5-phosphate kinase

by stromal metabolite levels. Biochim Biophys Acta 722, 51–60

Ghosh HP, Preiss J (1966) Adenosine diphosphate glucose pyrophosphorylase. A regulatory enzyme in the biosynthesis of starch in spinach leaf chloroplasts. J Biol Chem 241: 4491-4504

Giersch C, Heber U, Kaiser G, Walker DA, Robinson SP (1980a) Intracellular metabolite gradients and flow of carbon during photosynthesis of leaf protoplasts. Arch Biochem Biophys 205: 246-259

Giersch C, Heber U, Kobayashi Y, Inoue Y, Shibata K, Heldt HW (1980b) Energy charge, phosphorylation potential and proton motive force in chloroplasts. Biochim Biophys Acta 590: 59-73

Green DE, Leloir LF, Nocito, V (1945) Transaminases J. Biol. Chem.; 161, 559.

Gozzer C, Zanetti G, Galliano M, Sacchi GA, Minchiotti L, Curti B (1977) Molecular heterogeneity of ferredoxin-NADP+ reductase from spinach leaves. Biochim Biophys Acta 485: 278-290

Gustafson GL, Gander JE (1972) Uridine diphosphate glucose pyrophosphorylase from Sorghum vulgare. Purification and kinetic properties. J Biol Chem 247: 1387-1397

Hansen RG, Albrecht GJ, Bass ST, Seifert LL (1966) Methods Enzymol.; 8, 248

Harary I, Korey SR, Ochoa S (1953) Biosynthesis of dicarboxylic acids by carbon dioxide fixation. VII. Equilibrium of malic enzyme reaction. J Biol Chem 203: 595-604

Harbron S, Foyer C, Walker D (1981) The purification and properties of sucrose-phosphate synthetase from spinach leaves: the involvement of this enzyme and fructose bisphosphatase in the regulation of sucrose biosynthesis. Arch Biochem Biophys 212: 237-246

Hatch MD (1987) C-4 Photosynthesis - a Unique Blend of Modified Biochemistry, Anatomy and Ultrastructure. Biochim Biophys Acta 895: 81-106

Hatch MD, Burnell JN (1990) Carbonic-Anhydrase Activity in Leaves and Its Role in the First Step of C-4 Photosynthesis. Plant Physiol 93: 825-828

Hatch MD, Droscher L, Flugge UI, Heldt HW (1984) A Specific Translocator for Oxaloacetate Transport in Chloroplasts. Febs Lett 178: 15-19

Hawker JS, Ozbun JL, Ozaki H, Greenberg E, Preiss J (1974) Interaction of spinach leaf adenosine diphosphate glucose alpha-1,4-glucan alpha-4-glucosyl transferase and alpha-1,4-glucan, alpha-1,4-glucan-6-glycosyl transferase in synthesis of branched alpha-glucan.

Arch Biochem Biophys 160: 530-551

Hiraga K, Kikuchi G (1980) The mitochondrial glycine cleavage system. Purification and properties of glycine decarboxylase from chicken liver mitochondria. J Biol Chem 255: 11664-11670

Hoofd LJC, Tong RR, Stroeve P (1986) Nonequilibrium Facilitated Transport Of Carbon-Dioxide In Bicarbonate And Bovine Albumin Solutions. Ann Biomed Eng 14: 493-511

Howitz KT, McCarty RE (1985a) Kinetic characteristics of the chloroplast envelope glycolate transporter. Biochemistry 24:2645–52

Howitz KT, McCarty RE (1985b) Substrate specificity of the pea chloroplast glycolate transporter. Biochemistry 24: 3645–3650

Howitz KT, McCarty RE (1986) D-Glycerate transport by the pea chloroplast glycolate carrier. Studies on [1-14C]D-glycerate uptake and D-glycerate dependent O2 evolution. Plant Physiol 80: 390–395

Ireland RJ, Joy KW (1983) Purification and properties of an asparagine aminotransferase from Pisum sativum leaves. Archives of Biochemistry and Biophysics, 223, 291–296

Iwaki T, Wadano A, Yokota A, Himeno M (1991) Aldolase - an important enzyme in controlling the ribulose-l,5-bisphos- phate regeneration rate in photosynthesis. Plant Cell Physiol. 32, 1083-1091

Jang HK, Lee SW, Lee YH, Hahn TR (2003) Purification and characterization of a recombinant pea cytoplasmic fructose-1,6-bisphosphatase. Protein Expr Purif 28: 42-48

Jenkins CL, Burnell JN, Hatch MD (1987) Form of inorganic carbon involved as a product and as an inhibitor of c(4) Acid decarboxylases operating in c(4) photosynthesis. Plant Physiol 85: 952-957

Jenkins CL Hatch MD (1985) Properties and reaction mechanism of C4 leaf pyruvate,Pi dikinase. Arch Biochem Biophys 239: 53-62

Jenkins CL, Furbank RT, Hatch MD. (1989) Mechanism of C4 Photosynthesis - a Model Describing the Inorganic Carbon Pool in Bundle Sheath-Cells. Plant Physiol 91: 1372-1381

Julliard JH, Breton-Gilet A (1997) Identification of hydroxypyruvate reductase from parsley by peptide sequence comparison after a two-step purification. Protein Expr Purif 9: 10-14

Kagawa T, Bruno PL (1988) NADP-malate dehydrogenase from leaves of Zea mays: purification

and physical, chemical, and kinetic properties. Arch Biochem Biophys 260: 674-695

Keirns JJ, Wang JH (1972) Studies on nicotinamide adenine dinucleotide phosphate reductase of spinach chloroplasts. J Biol Chem 247: 7374-7382

Kerr MW, Gear CF (1974) Phosphoglycolate phosphatase isolated from pea leaves. Biochem. Soc. Trans. 2, 338-340

Kim Y, Yakunin AF, Kuznetsova E, Xu X, Pennycooke M, Gu J, Cheung F, Proudfoot M, Arrowsmith CH, Joachimiak A, Edwards AM, Christendat D (2004) Structure- and function-based characterization of a new phosphoglycolate phosphatase from Thermoplasma acidophilum. J Biol Chem 279: 517-526

Kleczkowski LA, Edwards GE (1989) Identification of hydroxypyruvate and glyoxylate reductases in maize leaves. Plant Physiol 91: 278-286

Kleczkowski LA, Randall DD (1988) Purification and characterization of a novel NADPH(NADH)-dependent hydroxypyruvate reductase from spinach leaves. Comparison of immunological properties of leaf hydroxypyruvate reductases. Biochem J 250: 145-152

Kleczkowski LA, Randall DD, Blevins DG (1986) Purification and characterization of a novel NADPH(NADH)-dependent glyoxylate reductase from spinach leaves. Comparison of immunological properties of leaf glyoxylate reductase and hydroxypyruvate reductase. Biochem J 239: 653-659

Kleczkowski LA, Randall DD, Zahler WL (1985) The substrate specificity, kinetics, and mechanism of glycerate-3-kinase from spinach leaves. Arch Biochem Biophys 236: 185-194

Kleczkowski, L. A., Villand, P., Lonneborg, A., Olsen, O. A. and Luthi, E. (1991). Plant ADP-glucose pyrophosphorylase--recent advances and biotechnological perspectives (a review). Z Naturforsch C 46: 605-612

Knaff DB (1996) Ferredoxin and ferredoxin-dependent enzymes. In DR Ort and CF Yocum, eds, Oxygenic Photosynthesis: The Light Reactions. Kluwer Academic Publishers, Dordrecht, The Netherlands, pp 333-361

Kochi H, Kikuchi G (1974) Mechanism of the reversible glycine cleavage reaction in Arthrobacter globiformis. I. Purification and function of protein components required for the reaction. J Biochem 75: 1113-1127

Kanai R, Edwards GE (1999) The Biochemistry of C4 Photosynthesis. In RF Sage, RK Monson,

eds, C4 Plant Biology. Academic Press, Toronto, pp 173–211.

Krebs HA (1953) Equilibria in transamination systems. Biochem. J.; 54, 82

Kretschmer M, Hofmann E (1984) Inhibition of rat liver phosphofructokinase-2 by phosphoenolpyruvate and ADP. Biochem Biophys Res Commun 124: 793-796

Kruger I, Schnarrenberger C (1983) Purification, subunit structure and immunological comparison of fructose-bisphosphate aldolases from spinach and corn leaves. Eur J Biochem 136: 101-106

Laisk A, Eichelmann H, Oja V, Eatherall A, Walker DA (1989) A mathematical model of the carbon metabolism in photosynthesis. Difficulties in explaining oscillations by fructose 2,6-bisphosphate regulation. Proc R Soc Lond B Biol Sci 237: 389–415

Laisk A, Edwards GE (2000) A mathematical model of C-4 photosynthesis: The mechanism of concentrating CO2 in NADP-malic enzyme type species. Photosynth Res 66: 199-224

Larondelle Y, Mertens E, Van Schaftingen E, Hers HG (1986) Purification and properties of spinach leaf phosphofructokinase 2/fructose 2,6-bisphosphatase. Eur J Biochem 161: 351-357

Lawson JWR, Guynn RW, Cornell N, Veech RL (1976) in "Gluconeogenesis: Its Regulation in Mammalian Species"; R.W. Hanson and M.A. Mehlman, eds.; John Wiley and Sons, New York. pp. 481-512.

Lazova GN, Stemler AJ (2008) A 160 kDa protein with carbonic anhydrase activity is complexed with rubisco on the outer surface of thylakoids. Cell Biol Int 32: 646-653

Leegood RC (1985) The Intercellular Compartmentation of Metabolites in Leaves of Zea-Mays-L. Planta 164: 163-171

Lemaire M, Miginiac-Maslow M, Decottignies P (1996) The catalytic site of chloroplastic NADP-dependent malate dehydrogenase contains a His/Asp pair. Eur J Biochem 236: 947-952

Li L, Preiss J (1992) Characterization of ADPglucose pyrophosphorylase from a starch-deficient mutant of Arabidopsis thaliana(L). Carbohydr Res 227: 227-239

Lunn JE, ap Rees T (1990) Apparent equilibrium constant and mass-action ratio for sucrose-phosphate synthase in seeds of Pisum sativum. Biochem J 267: 739-743

Lunn JE, Ashton AR, Hatch MD, Heldt HW (2000) Purification, molecular cloning, and sequence analysis of sucrose-6F-phosphate phosphohydrolase from plants. Proc Natl Acad Sci U S A 97: 12914-12919

Macdonald FD, Chou Q, Buchanan BB, Stitt M (1989) Purification and characterization of fructose-2,6-bisphosphatase, a substrate-specific cytosolic enzyme from leaves. J Biol Chem 264: 5540-5544

Macioszek J, Anderson LE (1987) Changing kinetic properties of the two enzymes phosphoglycerate kinase/NADP-linked glyceraldehyde-3-phosphate dehydrogenase couple from pea chloroplasts during photosynthetic induction. Biochem. Biophys. Acta 892:185–190.

Markham JE, Kruger NJ (2002) Kinetic properties of bifunctional 6-phosphofructo -2-kinase/fructose-2,6-bisphosphatase from spinach leaves. Eur J Biochem 269: 1267-1277

McGuire M, Carroll LJ, Yankie L, Thrall SH, DunawayMariano D, Herzberg O, Jayaram B, Haley BH (1996) Determination of the nucleotide binding site within Clostridium symbiosum pyruvate phosphate dikinase by photoaffinity labeling, site-directed mutagenesis, and structural analysis. Biochemistry-Us 35: 8544-8552

Moorhead GB, Plaxton WC (1990) Purification and characterization of cytosolic aldolase from carrot storage root. Biochem J 269: 133-139

Mukerji, SK (1977) Corn Leaf Phosphoenolpyruvate Carboxylases - Purification And Properties Of 2 Isoenzymes. Arch Biochem Biophys 182: 343-351

Nakamura, Y, Tolbert, NE (1983) Serine: glyoxylate, alanine:glyoxylate, and glutamate:glyoxylate aminotransferase reactions in peroxisomes from spinach leaves. J Biol Chem 258: 7631-7638

Nakano K, Omura Y, Tagaya M, Fukui T (1989) UDP-glucose pyrophosphorylase from potato tuber: purification and characterization. J Biochem 106: 528-532

Nilsson U, Hecquet L, Gefflaut T, Guerard C, Schneider G (1998) Asp477 is a determinant of the enantioselectivity in yeast transketolase. Febs Lett 424: 49-52

O'Leary MH, Reife JE, Slater JD (1981) Kinetic and isotope effect studies of maize phosphenolpyruvate carboxylase. Biochemistry 20:73 08--14

O'Leary MH (1984) Measurement of the isotopic fractionation associated with diffusion of carbon dioxide in aqueous solution. J. Phys. Chem. 88:823-25

Omnaas J, Porter MA, Hartman FC (1985) Evidence for a reactive cysteine at the nucleotide binding site of spinach ribulose-5-phosphate kinase. Arch Biochem Biophys 236: 646-653

Paszkowski A, Niedzielska A (1989) Glutamate:glyoxylate aminotransferase from the seedlings

of rye (Secale cereale L.). Acta biochimica Polonica 36: 17-29

Paszkowski, A, Niedzielska A (1990) Serine:glyoxylate aminotransferase from the seedlings of rye (Secale cereale L.). Acta biochimica Polonica 37: 277-282

Penefsky HS (1974) Mitochondrial and chloroplast ATPases. In Boyer, PD (ed.) The Enzymes. Academic Press, New York, pp. 375–395.

Pocker Y, Miksch RR (1978) Plant Carbonic-Anhydrase - Properties And Bicarbonate Dehydration Kinetics. Biochemistry-Us 17: 1119-1125

Porter MA, Milanez S, Stringer CD, Hartman FC (1986) Purification and characterization of ribulose-5-phosphate kinase from spinach. Arch Biochem Biophys 245: 14-23

Reger BJ, Ku MSB, Potter JW, Evans JJ (1983) Purification and Characterization of Maize Ribulose-1,5-Bisphosphate Carboxylase. Phytochemistry 22: 1127-1132

Reichert A, Baalmann E, Vetter S, Backhausen JE, Scheibe R (2000) Activation properties of the redox-modulated chloroplast enzymes glyceraldehyde 3-phosphate dehydrogenase and fructose-1,6-bisphosphatase. Physiol. Plant. 110, 330-341

Roeske CA, O'leary MH (1984) Carbon isotope effects on the enzymecatalyzed carboxylation of ribulose bisphosphate. Biochemistry 23:6275-84

Salerno GL, Pontis HG (1978) Studies on sucrose phosphate synthetase. The inhibitory action of sucrose. Febs Lett 86: 263-267

Schenk G, Duggleby RG, Nixon PF (1998) Properties and functions of the thiamin diphosphate dependent enzyme transketolase. The international journal of biochemistry & cell biology 30: 1297-1318

Schimkat D, Heineke D, Heldt HW (1990) Regulation of sedoheptulose-1,7-bisphosphatase by sedoheptulose-7-phosphate and glycerate, and of fructose-1,6-bisphosphatase by glycerate in spinach chloroplasts. Planta 181: 97–103

Schnarrenberger C, Kruger I (1986) Distinction between Cytosol and Chloroplast Fructose -Bisphosphate Aldolases from Pea, Wheat, and Corn Leaves. Plant Physiol 80: 301-304

Slabas AR, Walker DA (1976) Inhibition of spinach phosphoribulokinase by DL-glyceraldehyde. Biochem J 153: 613-619

Shin M (1972) Ferredoxin-NADPH reductase from spinach. Methods Enzymol. 23:440-445.

Son D, Jo J, Sugiyama T (1991) Purification and characterization of alanine aminotransferase

from Panicum miliaceum leaves. Arch. Biochem. Biophys. 289, 262-266

Sonnewald U, Quick WP, MacRae E, Krause KP, Stitt M (1993) Purification, cloning and expression of spinach leaf sucrose-phosphate synthase in Escherichia coli. Planta 189: 174-181

Sowinski P, Szczepanik J, Minchin PEH (2008) On the mechanism of C-4 photosynthesis intermediate exchange between Kranz mesophyll and bundle sheath cells in grasses. J Exp Bot 59: 1137-1147

Sowokinos JR (1981) Pyrophosphorylases in Solanum tuberosum: II. CATALYTIC PROPERTIES AND REGULATION OF ADP-GLUCOSE AND UDP-GLUCOSE PYROPHOSPHORYLASE ACTIVITIES IN POTATOES. Plant Physiol 68: 924-929

Sowokinos JR, Spychalla JP, Desborough SL (1993) Pyrophosphorylases in Solanum tuberosum (IV. Purification, Tissue Localization, and Physicochemical Properties of UDP-Glucose Pyrophosphorylase). Plant Physiol 101: 1073-1080

Sparla F, Fermani S, Falini G, Zaffagnini M, Ripamonti A, Sabatino P, Pupillo P, Trost P (2004) Coenzyme site-directed mutants of photosynthetic A4-GAPDH show selectively reduced NADPH-dependent catalysis, similar to regulatory AB-GAPDH inhibited by oxidized thioredoxin. J Mol Biol 340: 1025-1037

Sparla F, Zaffagnini M, Wedel N, Scheibe R, Pupillo P, Trost P (2005) Regulation of photosynthetic GAPDH dissected by mutants. Plant Physiol 138: 2210-2219

Speranza ML, Ferri G (1982) Glyceraldehyde-3-phosphate dehydrogenase (glycolytic form) from spinach leaves. Methods Enzymol. 89, 316-319

Sprenger GA, Schorken U, Sprenger G Sahm H (1995) Transketolase A of Escherichia coli K12. Purification and properties of the enzyme from recombinant strains. Eur J Biochem 230: 525-532

Stitt M, Heldt HW (1985) Generation and Maintenance of Concentration Gradients between the Mesophyll and Bundle Sheath in Maize Leaves. Biochim Biophys Acta 808: 400-414

Surek B, Heilbron A, Austen A Latzko E (1985) Purification and characterization of phosphoribulokinase from wheat leaves. Planta 165: 507-512

Tang GL, Wang YF, Bao JS Chen HB (2000) Overexpression in Escherichia coli and characterization of the chloroplast fructose-1,6-bisphosphatase from wheat. Protein Expr Purif 19: 411-418

Taniguchi M, Kobe A, Kato M, Sugiyama T (1995) Aspartate aminotransferase isozymes in Panicum miliaceum L., an NAD-malic enzyme-type C4 plant: comparison of enzymatic properties primary structures, and expression patterns. Arch. Biochem. Biophys. 318, 295-306

Teige M, Melzer M, Suss KH (1989). Purification, properties and in situ localization of the amphibolic enzymes D-ribulose 5-phosphate 3-epimerase and transketolase from spinach chloroplasts. Eur. J. Biochem. 252, 237-244

Tolbert NE (1981) Metabolic pathways in peroxisomes and glyoxysomes. Annu Rev Biochem 50: 133-157

Trost P, Scagliarini S, Valenti V, Pupillo P (1993) Activation of spinach chloroplast glyceraldehyde 3-phosphate dehydrogenase: Effect of glycerate 1,3-bisphosphate. Planta 190: 320–326

Uchida K, Mochizuki M, Niizeki K (1983) Diffusion-Coefficients Of Co2 Molecule And Bicarbonate Ion In Hemoglobin Solution Measured by Fluorescence Technique. Jpn J Physiol 33: 619-634

Uedan, K, Sugiyama T (1976) Purification And Characterization Of Phosphoenolpyruvate Carboxylase From Maize Leaves. Plant Physiol 57: 906-910

Uehlein N, Otto B, Hanson DT, Fischer M, McDowell N, Kaldenhoff R (2008) Function of Nicotiana tabacum aquaporins as chloroplast gas pores challenges the concept of membrane CO2 permeability. Plant Cell 20: 648-657

Van RC, Pan YJ, Hsu SH, Huang YT, Hsiao YY, Pan RL (2005) Role of transmembrane segment 5 of the plant vacuolar H+-pyrophosphatase. Biochim Biophys Acta 1709: 84-94

Villadsen D, Nielsen TH (2001) N-terminal truncation affects the kinetics and structure of fructose-6-phosphate 2-kinase/fructose-2,6-bisphosphatase from Arabidopsis thaliana. Biochem J 359: 591-597

von Caemmerer S (2000) Biochemical models of leaf photosynthesis. CSIRO Publishing, Collingwood.

von Caemmerer S, Furbank RT (2003) The C-4 pathway: an efficient CO2 pump. Photosynth Res 77: 191-207

Walker GH, Huber SC (1987) ATP-dependent activation of a new form of spinach leaf 6-phosphofructo-2-kinase/fructose 2,6-bisphosphatase. Arch Biochem Biophys 258: 58-64

Whitaker DP (1984) Purification and properties of sucrose-6-phosphatase from Pisum sativum shoots. Phytochemistry 23, 2429–2430.

Winter H, Robinson DG, Heldt HW (1993) Subcellular Volumes And Metabolite Concentrations In Barley Leaves. Planta 191: 180-190

Woodrow IE, Walker DA (1983) Regulation of stromal sedoheptulose 1,7-bis- phosphatase activity and its role in controlling the reductive pentose phosphate pathway of photosynthesis. Biochim Biophys Acta 722:508-516.

Woodrow IE, Mott KA (1993) Modeling C3 photosynthesis—a sensitivity analysis of the photosynthetic carbon reduction cycle. Planta 191: 421–432

Xu W, Ahmed S, Moriyama H, Chollet R (2006) The importance of the strictly conserved, C-terminal glycine residue in phosphoenolpyruvate carboxylase for overall catalysis: mutagenesis and truncation of GLY-961 in the sorghum C4 leaf isoform. J Biol Chem 281: 17238-17245

Yu S, Xia D, Luo Q, Cheng Y, Takano T, Liu S (2007) Purification and characterization of carbonic anhydrase of rice (Oryza sativa L.) expressed in Escherichia coli. Protein Expr Purif 52: 379-383

Zelitch I (1955). Glycolic acid oxidase and glyoxylic acid reductase. In SP Colowick, NO Kaplan, eds, Methods in Enzymology, Vol I. Academic Press, New York-London, pp 528-535

Zhu X G, De Sturler E, Long SP (2007) Optimizing the distribution of resources between enzymes of carbon metabolism can dramatically increase photosynthetic rate: A numerical simulation using an evolutionary algorithm. Plant Physiol 145: 513-526

Ziegler I (1974) Malate dehydrogenase in Zea mays: properties and inhibition by sulfite. Biochim Biophys Acta 364: 28-37
